# Maternal health care-seeking behaviour of married adolescent girls: A prospective qualitative study in Banke District, Nepal

**DOI:** 10.1371/journal.pone.0217968

**Published:** 2019-06-25

**Authors:** ASM Shahabuddin, Therèse Delvaux, Christiana Nöstlinger, Malabika Sarker, Azucena Bardají, Alyssa Sharkey, Ramesh Adhikari, Sushil Koirala, Md Asadur Rahman, Tahrima Mridha, Jacqueline E. W. Broerse, Vincent De Brouwere

**Affiliations:** 1 Health Section, United Nations Children’s Fund (UNICEF), New York, New York, United States of America; 2 Department of Public Health, Institute of Tropical Medicine, Antwerp, Belgium; 3 James P Grant School of Public Health, BRAC University, Dhaka, Bangladesh; 4 ISGlobal, Barcelona Centre for International Health Research (CRESIB), Hospital Clínic-Universitat de Barcelona, Barcelona, Spain; 5 Geography and Population Department, Tribhuvan University, Kathmandu, Nepal; 6 Damien Foundation, Kathmandu, Nepal; 7 BRAC, Dhaka, Bangladesh; 8 Athena Institute for Research on Innovation and Communication in Health and Life Sciences, VU University, Amsterdam, The Netherlands; University of British Columbia, CANADA

## Abstract

**Background:**

Nepal has one of the highest rates of maternal mortality in the South Asia region, partly due to the underutilization of maternal health services and the high number of adolescent pregnancies. This study explores married Nepali adolescent girls’ healthcare-seeking behaviour throughout their pregnancies, during their delivery and postpartum.

**Methods:**

We conducted a prospective qualitative study in Banke district, Nepal. In-depth interviews were conducted with 27 married adolescent girls before and after delivery. In addition, a focus group discussion was conducted with community health works and key-informant interviews were conducted with family members of adolescent girls, representatives from the government and health care providers. We applied the Social-Ecological Model (SEM) as a framework to guide thematic content analysis and presentation of our qualitative data.

**Results:**

Several factors in the SEM influenced maternal health care-seeking behaviour of adolescents. At the individual level, girls’ perceptions, their lack of knowledge about maternal and reproductive health, certain traditional practices, their sole dependency on their husbands and mothers-in-laws and their low decision-making autonomy towards their own health care negatively influenced their utilization of skilled maternal health services. Mothers-in-law and other family members played a critical role in either encouraging or discouraging the use of skilled maternal health services. At the health systems level, lack of adolescent-friendly maternal health services, difficulties in accessing quality maternal health services, and the fixed operating hours of public health facilities restricted their ability to obtain services. The existence of the Safe Motherhood Programme, knowledge sharing platforms such as “women’s groups” and the active role of Female Community Health Volunteers (FCHVs) positively influenced utilization of skilled maternal health services among these girls.

**Conclusion:**

Influences on married adolescent girls’ use of skilled maternal health services in Banke District, Nepal were multi-factoral. Ensuring easy access and availability of adolescent-friendly maternal health services are important to encourage adolescent girls to use skilled maternal health services. Moreover, interventions are needed to improve adolescent girls’ knowledge of maternal health, keep them in school, involve family members (mainly mothers-in-law) in health interventions, as well as overcome negative traditional beliefs within the community that discourage care-seeking for skilled maternal health services.

## Introduction

Childbirth during adolescence (aged 10–19 years) is associated with severe health and social consequences. However, every year about 16 million adolescent girls between the ages of 15–19 give birth, of which about 95% occur in low-income countries (LICs) [1].

In many LICs, complications during pregnancy and childbirth are the leading causes of deaths among adolescents [2,3]. Adolescent mothers often suffer from anaemia, eclampsia, postpartum haemorrhage and mental disorders, such as depression [3–7]. Literature also showed that perinatal deaths are 50% higher among babies born to mothers under 20 years of age compared to those born to mothers aged 20 to 29-years-old [1,8]. Furthermore, babies born to adolescent girls often have low birth weight and long-term complications.

Timely access to skilled maternal health services is necessary to decrease the rates of adverse maternal health outcomes among both women and adolescents [6,8, 9–13]. Nepal has the second highest rate (17%) of adolescent pregnancy in South Asia [14]. Like many other countries in the region, child marriage is a deeply rooted societal norm, which has led to a high rate of adolescent pregnancies. Although the legal age of marriage in Nepal (with a guardian’s consent) is 18, about 37% of females in Nepal get married before that age [14,15].

Since 2009, the government of Nepal has provided facility-based deliveries free of charge to incentivize women to deliver in health facilities. However, Nepal continues to have the second highest maternal mortality ratios (MMR) in the region (229 deaths per 100,000 live births) [16,17], with underutilization of maternal health services a contributing factor [18]. The most recent demography and health survey conducted in 2016, showed that about 84% of pregnant women received antenatal (ANC) care from a skilled provider, while only 57% of childbirths occurred in health institutions [14]. In the case of adolescent girls, about 37% of deliveries occur at home, many of which (36%) are done without skilled birth attendants [14].

To improve maternal health, it is important that all women give birth with skilled personnel. Adolescent maternal health care-seeking behaviour is a complex behavioural phenomenon, yet one which is critical to improving healthcare outcomes [6, 19]. Several quantitative studies conducted in Nepal have identified that age, education, employment status, location of the residence and parity influenced use of maternal health services among women of reproductive age [12, 20–23]. To date, there are no published qualitative studies which explicitly explored other aspects (interpersonal, community and health systems) of maternal health care-seeking behaviour of married adolescents in Nepal. Therefore, this study aimed to explore the health care-seeking behaviour of married adolescent girls in Nepal during pregnancy, delivery and post-delivery. Using qualitative methods, this study explicitly explored factors influenced utilization of maternal health services among married adolescent girls in Banke district, Nepal.

## Materials and methods

### Study design

This study was part of a larger qualitative study conducted in Bangladesh and Nepal. We conducted a two-phase prospective qualitative study among married adolescent girls (both married pregnant and married non-pregnant) in Nepal. During the baseline data collection phase, we explored married adolescent girls’ knowledge, perceptions, and intentions regarding the use of maternal health services. After 16 months, during the follow-up phase, we explored their actual behaviours and explanations towards their use (or non-use) of maternal health services including antenatal, delivery and postnatal care (PNC). In-depth interviews (IDIs), key informant interviews (KIIs), and a focus group discussion (FGD) were conducted with different stakeholders to triangulate and validate the data.

### Study setting

The study was conducted in the Banke district of Nepal which has a population of around 500,000. Banke district is within the Terai (low plains) area of Nepal,and situated in the Bheri zone of the Mid-Western region. Nepalgunj acts as its administrative headquarters. The district itself is close to the Indian border with Uttar Pradesh being the nearest sub-division of India. The major ethnic groups in the district include Tharu, Musalman (Muslim), Chhetri and Brahman. However, native Nepalese living in the Terai region are called ‘Madhesi’ [24].

There are different levels of health facilities through which the government of Nepal provides basic and emergency health care services. The ‘Sub-Health Post’ is the first level to provide basic health care services, including general health, immunization and vaginal delivery care services. Health posts are the next level of care and also provide ANC and vaginal delivery care. In addition, there are three PHCs (Primary Health Centre) and two large hospitals in the district of Banke, all of which provide maternal health services including caesarean sections. The government district hospital, named Bheri Zonal Hospital, is situated in Nepalgunj. Additionally, there is a private teaching hospital in Nepalgunj named Nepalgunj Medical College. According to the 2011 Nepal DHS (Demographic and Health Survey), the Mid-Western region has the highest rate (21%) of adolescent pregnancy in Nepal [16].

### Study population

A range of adolescent married girls was sampled to achieve variation in location, socio-economic background, age group and educational background. In addition, we collected data from community health workers, family members of adolescent girls (mothers-in-law and husbands), government representatives and health care providers. Married adolescent girls and other categories of respondents were selected purposively with the help of Female Community Health Volunteers (FCHVs) and representatives of TB Nepal (a local non-governmental organization). Table 1 shows a list of study participants and data collection methods.

**Table 1:** Data collection methods and study participants in baseline and follow-up phase.

Methods	Participants	
	Baseline (August 2014)	Follow-up phase (December 2015)
In-depth interviews (IDIs)	Married pregnant adolescent girls (n = 22)	Same girls from phase 1 (n = 18) [lost to follow-up, n = 4]
	Married non-pregnant adolescent girls (n = 10)	Same girls from phase 1 (n = 9) [lost to follow-up, n = 1] Husbands of adolescent girls (n = 1) Mothers-in-law (n = 2)
Focus group discussion (FGD)	Community health workers (FCHVs) (n = 7)	
Key informant interviews (IDIs)	Government health officer (District Public Health Officer in Banke) (n = 1) In-charge of a health post (n = 1) Medical doctor (gynaecologist) in Bheri zonal hospital (n = 1)	

### Data collection

In-depth interviews (IDIs) were conducted with married pregnant and non-pregnant adolescent girls in two phases. At baseline (August 2014), pregnant adolescents were asked about their knowledge, perceptions and practices related to maternal health care services and their intended plan for delivery, while non-pregnant adolescents were interviewed about their knowledge, perception and practices related to family planning methods and their childbearing intentions. During the follow-up phase, (conducted in December 2015–16 months after the first phase), the same participants were asked whether they had become pregnant, and if so, were then asked about their experiences with ANC, delivery services and PNC. For both groups, the information collected in the baseline and the follow-up phase helped to capture a broader picture of maternal care-seeking behaviour.

KIIs were conducted with government health officials and health care providers to explore issues related to socio-cultural beliefs and practices influencing adolescent girls’ use of contraceptive methods and skilled maternal health services. One FGD was conducted with community health workers (locally called Female Community Health Volunteers in a health post of Khajura VDC. Interviews with adolescent girls and their mothers-in-law were conducted in Nepali by female Nepali researchers (with nursing backgrounds and could speak Nepali and Hindi) who were trained prior to data collection. Interviews with adolescent girls were conducted at their residence. A few interviews were conducted in Hindi, particularly with Muslim adolescent girls who did not speak Nepali. The first author (ASMS) and a Nepali researcher (SK) conducted key informant interviews and FGD with health care providers. Each IDI and KII took about 40 minutes to complete while the duration of FGD was about an hour. The interview guides were pre-tested in Nepalgunj, Banke district and adapted accordingly. All topic guides were developed in English and then translated into Nepali before pretesting. All interviews were audio recorded, transcribed verbatim and translated in English by Nepali researchers.

### Data analysis and theoretical framework

**Fig 1:**
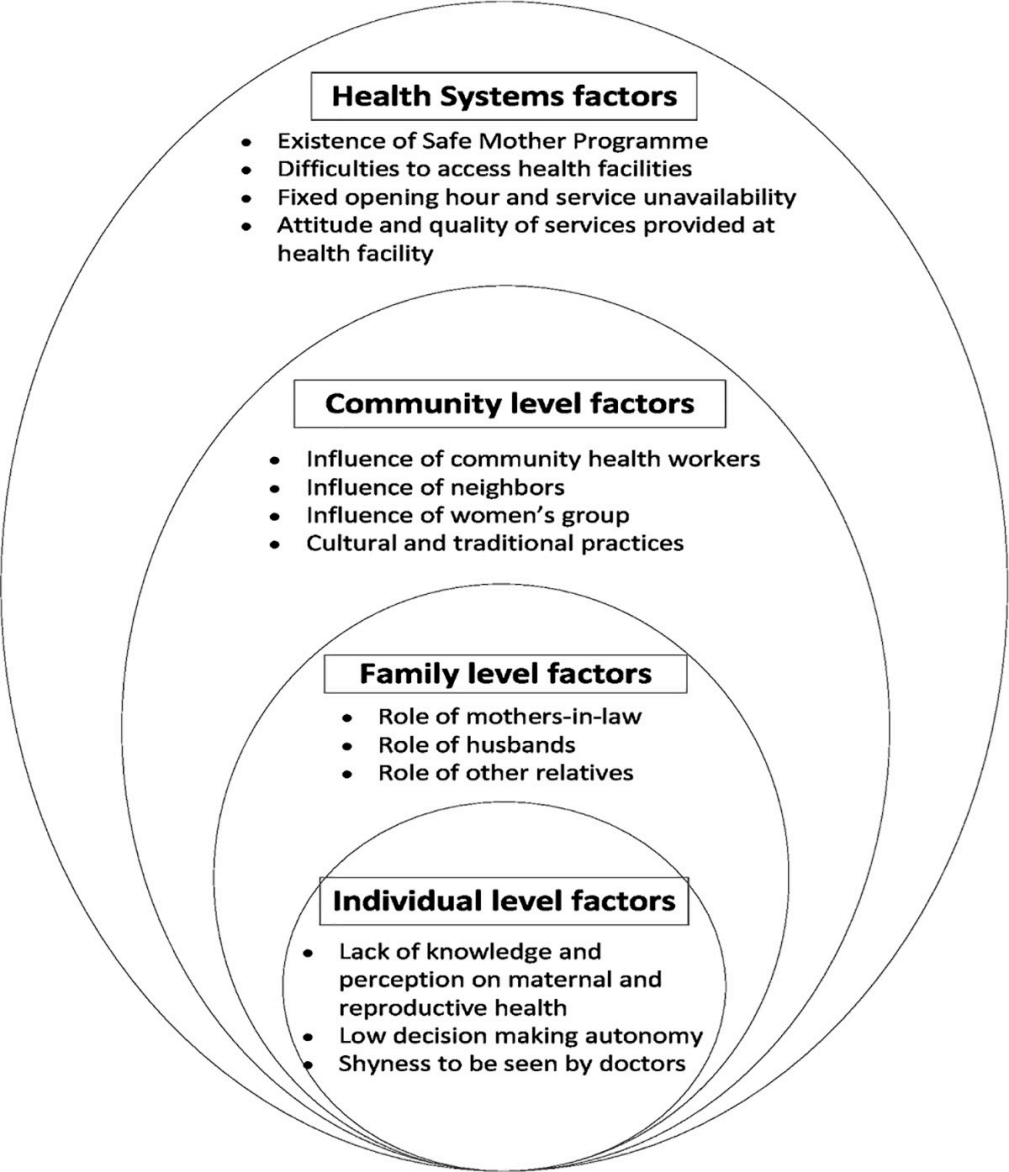
Four level of factors in SEM which influenced maternal health care-seeking behaviour among adolescents in Nepal.

Thematic analysis was done which was guided by the Social-Ecological Model (SEM) [25, 26]. SEM was used as an analytical framework to develop an initial coding guide during data analysis. The SEM is a theory-based framework that considers the complex interplay of multiple levels of a social system, including the interactions between individuals and the environment within the system. The SEM facilitated the exploration of adolescent girls’ experiences, the influences of their families, and the community and socio-cultural contexts that all work on different levels to influence maternal health care-seeking behaviours. Guided by the objectives of the study and the SEM, an initial coding framework was generated first author (ASMS) and two co-authors (TD, CN) after reading a subset of the transcripts. Newly emerging codes, from subsequent transcripts were inductively added to the framework to build our model of factors or themes influencing maternal health care-seeking behaviour (Fig 1). When new codes and themes were added to the framework, all data were re-evaluated to assess their relevance through constant comparison. The data from IDIs, KIIs and the FGD were analysed several times to obtain a sense of how these data contributed to the framework as a whole. To increase the validity of our data, researchers (first author and three co-authors) with different backgrounds (i.e. social science, anthropology, medical science) read majority of the transcripts and provided input to the analysis process.

### Ethical considerations

The qualitative study protocol was approved (no. 943/14) by the Institutional Review Board (IRB) of the Institute of Tropical Medicine (ITM) in Antwerp, Belgium. Ethical approval was also obtained from the Nepal Health Research Council (NHRC- reg. no. 121/2014). Almost all study participants provided written informed consent by signing the informed consent form. The few participants who did not feel comfortable providing a signature on the informed consent form were permitted to give verbal consent. This process was witnessed by a third party (i.e. family member or another researcher). Participants below the age of 18 provided assent and written consent was obtained from their legal guardians or husbands. Confidentiality was strictly maintained throughout the study and only the researchers had access to the data. No personal identifying information was retained that could identify the participants.

## Results

We conducted a total of 32 interviews at baseline in August 2014, of which 22 interviews were with pregnant adolescent girls and 10 were with non-pregnant adolescent girls. During the follow-up phase of data collection (December 2015), we re-interviewed 27 adolescent girls of the previous 32 participants. We were not able to re-interview five girls, because of the change of their residence or due to their travels. Among 18 adolescent pregnant girls who participated in both phases of data collection, one had a neonatal death and one experienced a stillbirth. Among the 9 non-pregnant adolescents interviewed at baseline, at follow-up three had become mothers, two were pregnant and another girl experienced a still-birth (Table 2).

**Table 2:** Characteristics of the adolescent girls during baseline and follow-up interviews.

Characteristic of the adolescent girls during baseline (August 2014)	Characteristics during follow-up phase (December 2015)					
	Had a baby	Became pregnant	Not yet pregnant	Neonatal death	Stillbirth	Total
Pregnant	16	0	0	1	1	18
Not-pregnant	3	2	3	0	1	9
**Total**	**19**	**2**	**3**	**1**	**2**	**27**

### Intended place of delivery and actual experiences

During the first phase of data collection, we asked pregnant adolescent girls where they intended to deliver their babies and we asked non-pregnant adolescent girls about their intended place of delivery should they become pregnant. Ten adolescent girls who said that they wanted to deliver their babies in the hospital successfully did so. Of the four girls who reported wanting to deliver at home, two delivered in health facilities. Seven girls did not have any plans for the location of their deliveries. Among those seven girls, one girl delivered at home and the other six delivered at the health facility (Table 3).

**Table 3:** Delivery intention and actual place of delivery.

Intended place of delivery	Actual place of delivery		Total
	Home	Health facility	
Home	2	2	4
Health facility	0	10	10
Not sure	1	6	7
**Total**	**3**	**18**	**21**

### Characteristics of the adolescent girls and their use of maternal health services

We collected data on the knowledge and use of maternal health services (ANC, place of delivery and PNC services) from the adolescent girls who participated in both phases of the research. Ten girls were aged between 14–17 years old, while fifteen were 18–19 years old. Fifteen out of the 24 girls received less than four ANC visits, three out of 21 girls delivered at home, while 15 girls delivered in a public health facility, and three in a private clinic or hospital. Five of the adolescent girls had caesarean sections. Among all 21 girls who had given birth, 15 did not receive PNC (Table 4).

**Table 4:** Characteristics of the participants and use of maternal health services among adolescent girls in Banke, Nepal (n = 21 for delivery and PNC; n = 24 for ANC).

Characteristics	Uptake of 4 ANC visits with qualified provider		Place of delivery			Mode of delivery		PNC from qualified provider	
	Yes	No	Home	Facility (public)	Facility (private)	Vaginal	CS*	Yes	No
**Age**									
14–17 years	4	6	2	6	1	8	1	1	8
18–19 years	5	9	1	9	2	8	4	5	7
**Village Development Committee (VDC)**									
Khajura	5	6	1	8	1	7	3	4	6
Noubasta	2	3	1	2	2	3	2	2	3
Sitapur	1	1	0	1	0	1	0	0	1
Udarapur	1	2	1	2	0	3	0	0	3
Bageswari	0	3	0	2	0	2	0	0	2
**Education of the girl**									
No formal education	0	2	1	1	0	2	0	0	2
Primary school or Less	2	1	0	2	0	2	0	0	2
Secondary school (any)	7	11	2	11	3	12	4	5	11
Higher than Secondary	0	1	0	1	0	0	1	1	0
**Total**	**9**	**15**	**3**	**15**	**3**	**16**	**5**	**6**	**15**

*CS: Caesarean section

Note: A Village Development Committee (VDC) in Nepal is the lowest administrative region of its Ministry of Federal Affairs and Local Development. Each district has several VDCs, which are similar to municipalities but with greater public-government interaction and administration.

### Social-ecological model to explore maternal health care-seeking behaviour

Using the Social-Ecological Model, we present the study findings within four broad categories that were found to influence the health care-seeking behaviour of adolescent girls in this study (Fig 1).

### Individual level factors

**Knowledge and perception of pregnancy and reproductive health.** Most of the adolescent girls (n = 19) were aware that pregnancy during adolescence was a risk both for the babies and the mothers, as illustrated by the following quotes:

‘*I don’t want a baby right now*. *I am still very young and this is not an appropriate age to give birth to a child’- (19-year-old non-pregnant girl*, *baseline*, *IDI)*

*‘If you get married at a very young age*, *then your body is not physically fit and mature enough to have a child’- (18-year-old girl*, *who had a still birth*, *follow-up phase*, *IDI)*.

However, out of 9 non-pregnant married adolescent girls interviewed during baseline we found 5 of them became pregnant (3 girls already gave birth) during the follow-up study. Despite their knowledge of the adverse effects of adolescent pregnancy, these girls were unsuccessful at avoiding pregnancy.

**Knowledge and perception about maternal health**. Most adolescent girls (n = 18) reported that ANC visits, hospital deliveries and PNC visits were important, although 15 girls did not receive adequate (at least four) ANC or any PNC.

*‘It is safe to go to health clinics as the doctors have better knowledge but if delivered in the home then they don’t know much and they forcefully pull the baby out’ (18-year-old girl who delivered in a health post*, *follow-up phase*, *IDI)*

There were a few girls who did not acknowledge the importance of using skilled maternal health services. When we asked about the importance of check-ups during pregnancy, a 16-year old pregnant adolescent girl said:

*‘I don’t think it is important*. *I think it is necessary to go for a check-up when the menstruation stops’- (16-year-old pregnant girl at follow-up interview*, *IDI)*

A government officer in KII mentioned the lack of awareness among the community and lack of knowledge about ANC and PNC check-ups. He also reported that women are reluctant to share information regarding their pregnancies.

*‘The main reason behind this [not using ANC] is lack of awareness in the society and lack of knowledge that one should have timely check-ups*. *Another reason is people are reluctant to share that they have a missed period or that they are pregnant*. *This is also the reason why they are late for their first ANC visit itself and also for less frequent 4*^*th*^ *ANC visit’- (Government health officer*, *KII)*

**Decision-making autonomy**. Only three adolescent girls mentioned that they participated in household decision-making, including the use of health care. Decisions were reportedly made either by husbands or by mothers-in-law. When we asked about the decision to become pregnant, a girl replied:

*‘No*, *I didn’t consult with anyone*. *I was willing to have a child so I consulted with my husband and decided on having only one child so we won’t be having any other child now’- (19-year-old during second phase*, *IDI)*

The majority of girls indicated they had very little decision-making autonomy towards their own health care, including maternal health. During the baseline, we asked an 18-year old girl about her intention for delivery, she replied;

*‘I don’t know*, *this is the first time I am pregnant*. *Maybe my mother-in-law will decide it*.*’*

When we asked about the four ANC visits, one of the adolescent girls replied:

*‘I didn't have the guts to tell my family members (mainly her in-laws) about it’-(17- year-old girl who had a still birth*, *first phase*, *IDI)*

**Shyness to be seen by men.** Shyness was mentioned as a reason for home delivery, as there were male healthcare workers at the health facility. An adolescent girl said that if female healthcare workers were assisting her at delivery she would be prepared to deliver her baby at a hospital. She mentioned,

*‘Because nurses are 'ladies'*, *and we are also ladies and I do not feel shy with the ladies*. *But I do feel shy in front of males'- (16-year-old non-pregnant girl*, *first phase*, *IDI)*

Another girl mentioned that girls often want to deliver at home, within their families, since there would be strangers at the hospital.

*‘They (girls) feel shy to get naked in front of everyone at the health post during delivery*. *But at home the people are familiar to her*, *she knows them’- (18 years old girl*, *first phase*, *IDI)*

### Factors related to interpersonal or family members and relatives

Adolescent girls’ in-laws play an important role in influencing their use of skilled maternal health services. Mothers-in-law were considered as the most experienced persons to share advice about maternal health issues. Thus, their decisions about maternal health services were often heeded.

*‘Both I and my mother in law are women*. *Who else would understand what I am going through better than her*? *That's why she decides it’- (17-year-old girl*, *delivered at home*, *first phase*, *IDI)*

One of the community health workers also mentioned the role of family members in making decisions related to ANC:

*‘Yesterday we organized a program but only 5 or 6 pregnant women attended the program and we go to the house of the one who didn’t and we personally talked to them and their family members that they should attend such programs*. *Some of them do not come with their own will and some do not come*, *as they are not allowed by their families’ (a female community health volunteer*, *in FGD)*

Another girl said that her older sister took her to the health facility. A few girls mentioned the role of their husband in this regard.

*‘Because my sister took me to the health centre for a check-up*. *And everyone goes to get a check-up these days so I also went there*.*’- (17-year-old girl*, *second phase*, *IDI)*

### Community level factors

**Role of community health workers.** Female community health volunteers played a very important role in encouraging pregnant girls to seek healthcare facilities both for their ANC visits and for delivery. Most of the women said that they received information about maternal health from the FCHV. Often, a FCHV visits the house of a newly pregnant woman in the village and provides information about the importance of ANC, PNC and deliveries at health facilities.

*‘There are FCHVs in the village*, *one of them told me to get a check-up done and I did’-(18 years old girl*, *delivered in a health post*, *follow-up phase*, *IDI)*

One FCHV mentioned that they organized an awareness-raising program in the village, which encouraged pregnant women to seek ANC and choose to deliver at healthcare facilities.

*‘We organize a program where we give health education and health promotional activities*. *Who attends our program comes for regular health check-ups but those who do not attend the program come for the check-up very late’- (a FCHV in FGD)*

**Role of neighbours.** Pregnant adolescent girls and their pregnant neighbours often go to the nearest health facility for their ANC visits together. When the health facility is far from the village, pregnant women prefer to visit the health facility together with their neighbours.

*‘I had a pregnant neighbour with close delivery dates*. *We would go together’-(17- year-old girl delivered in a health facility*, *IDI)*

Moreover, neighbours also played an important role as the source of information about healthcare facilities and other maternal health-related information. As one of the girls mentioned:

*‘Neighbours used to tell and I used to hear about check-up during pregnancy as well thus*, *I myself knew about it’-(19-year-old girl*, *who delivered in a private clinic*, *follow-up phase*, *IDI)*

**Existence of a women’s group.** Some girls (n = 8) mentioned that they came to know about the importance of ANC visits and delivery care through attending the women’s group meeting organized by FCHV.

*‘We have a women's group and we sit and have a discussion*. *I came to know about this from the group’-(15-year-old girl*, *baseline*, *IDI)*

**Cultural practices.** Cultural practices, rooted in the community, influenced the girls’ decisions not to seek PNC care. Women from the Chaudhary community (Tharu community), in particular, do not bring new-borns out of the home during their first 10 days of life, which restricts the use of PNC care according to the prescribed schedule in Nepal (within 24 hours of birth, on the third day and on the seventh day of the new-born’s life).

*‘Most of the people living in this VDC are Chaudhary communities*. *In the Chaudhary community*, *there is a religious and cultural restriction as the mothers are not allowed to come out of the house for 10 days after delivery*. *So*, *the mothers just remain at their home’-(Government officer*, *KII)*

### Health systems level factors

**Safe Motherhood Programme**. Through the ‘Safe Motherhood Programme’, the government of Nepal provides cash incentives to women who use maternal health services, including ANC, facility delivery and PNC. The girls reported that these incentives encouraged them to seek skilled maternal health services.

*‘Due to the monetary service that the government hospitals provide after a delivery*, *they wish to go to the hospital*, *in Bheri*. *They give NPR 500–1000; it’s 1000 at the hilly region*, *but 500 in Terai*. *So*, *in seek of that remuneration*, *people go to the hospital’- (18-year-old girl*, *delivered in a health facility*, *follow-up phase*, *IDI)*

Despite delivering at home, one girl reported later going to the health facility to receive money and baby clothes.

*‘It is easy to have the child delivered at the health post*. *They also give money to the mother and clothes to the child at the health post*. *That's why’–(17-years-old girl delivered at home*, *IDI)*

**Access to public health facilities.** Most adolescent girls reported difficulties in accessing public healthcare facilities. Most girls used bicycles to get to the healthcare centres for ANC, delivery and PNC, while other girls walked.

*‘The availability of vehicles is a difficulty as it is hard to travel on a bicycle*. *That’s it*, *what else do I say*? *We have to walk and that’s hard because of lack of transportation*. *This is how it is in village areas’-(18-year-old girl*, *delivered in a health facility*, *follow-up phase*, *IDI)*

In some areas, bullock-cart and motorcycles were also used as transport to the health facility. During the monsoon, deteriorating road conditions restricted pregnant girls from coming to the health facility for maternal health services.

*‘But during monsoon season*, *when the road access is halted*, *the women cannot come to the health centre*. *For such situation*, *we have given all the pregnant women our telephone number*. *We have told the women to call us immediately in case of any emergency or problems’-(In-charge of a health post*, *KII)*

In some emergency cases, pregnant girls were taken to the health centres using an ambulance. Each pregnant woman receives about 500 Nepali Rupees ($5) for delivering in a public health facility. However, as some participants mentioned, that amount is not sufficient to cover ambulance services.

**Service unavailability and fixed opening hours of the health facility.** Two adolescent girls mentioned the health post and sub-health post’s inflexible opening hours, which are the first point of contact for pregnant girls for ANC or any pregnancy-related issues.

*‘The opening time is 10 AM and the closing time is 5 PM*. *If you go between that times*, *you can get checked or you can’t before 10 AM or after 5 PM*. *That’s a problem*. *Sometimes you can spare time and sometimes you cannot so there’s difficulty’-(16-year-old non-pregnant adolescent*, *follow-up phase)*

Other girls also reported that the lack of available maternal health services and skilled health care providers at the public health facility precluded them from going to the hospital.

**Attitude of the health care providers.** Some participants mentioned that the lack of empathy from midwives and doctors at the hospital also impeded them from going to the health facility. Two adolescent girls said that they were scared of the health care workers and the delivery process. They did not want to go to the hospital because they feared that the nurses at the hospital would be rude and unsympathetic.

*‘Some pregnant women who already have many children hesitate to go to the health centre because they are scared that the doctors might scold them for having too many children*. *I know an aunt*, *who already had many children who were still very small*, *but she was again pregnant with another*. *When she visited the health centre*, *she got a scolding from the doctor*. *She never went to the health centre for check-up after that incident–(18-year-old girl delivered in a health facility*, *IDI)*

**Quality of the services at public health facilities.** A few participants brought up the poor quality of the public hospitals as a reason for choosing to deliver at a private health facility. When asked about going to the private health facility for delivery care, a girl replied:

*‘Because at the government hospitals*, *they do not really care about the patients and the small hospitals don't provide good services*. *Only big private hospitals care for the patients*. *I don't know the reason behind it but whenever we go to the hospital*, *I don't feel like they care much for us*. *The attitude of doctors and nurses there also isn't proper while talking to us*.*’ (17-year-old girl*, *baseline*, *IDI)*

## Discussion

The study found that several factors of each level of SEM negatively impacted the maternal health care-seeking behaviour of adolescent girls. Individual level factors, such as perceptions, in addition to lack of knowledge about maternal and reproductive health, low decision-making autonomy, shyness toward male service providers and fear of health care providers more generally, were perceived as barriers by adolescent girls in seeking ANC, PNC and hospital deliveries. Mothers-in-law and other family members (i.e. sisters-in-law) played a critical role in either encouraging or discouraging the use of skilled maternal health services. In the case of community level factors, CHWs, neighbours and women’s groups positively influenced the use of skilled maternal care. Moreover, non-availability of adolescent-friendly maternal health services, difficulties in accessing the services were they available, poor attitudes of providers and poor quality of care were also important impediments to health service utilization of the study participants.

The girls with low levels of education had limited knowledge about reproductive health, including the importance of skilled maternal health services. Education is an important factor necessary to understanding the need for skilled health care during pregnancy, which has also been shown in other studies which identified empowerment and independence as determinants in seeking maternal health services and institutional deliveries [27]. In other studies, female education had a positive association with facility use, while women who delivered at home had a lower level of education [28,20–21, 29], and vice versa, that poor maternal education increased the likelihood of home delivery [22,23]. Similar studies found that female education and knowledge about sexually transmitted diseases such as HIV/AIDS significantly increased the use of maternal health services [30].

Child marriage is directly related to girls dropping out from schools. When adolescent girls drop out of school, they are exposed to higher risk of domestic violence and abuse, increased economic dependence, and are more likely to be denied decision-making power [3,31]. We found that most of the adolescent girls knew about the importance of ANC, hospital delivery and PNC but many of them did not receive adequate (minimum eight ANC) ANC or PNC due to their low decision-making autonomy. Autonomy can be described as an individual’s ability to use the resources and information available to him or her to make decisions about one's own concerns [32–34]. Decision-making autonomy is an individual-level factor that can have an impact on health and is dependent on the aforementioned individual and interpersonal-level factors, including age, girls’ knowledge of maternal health, their interpersonal relationships and the influence of their mothers-in-laws, their community, as well as their cultural practices. Due to the girls’ low educational levels, their young age and financial dependency, they were unable to make their own autonomous decisions, including decisions about their own bodies and their baby’s, which instead comes under the purview of other family members. A study conducted in Bangladesh showed that women with average decision-making autonomy had more deliveries assisted by a trained provider than women who had low autonomy [35].

Interpersonal factors in the personal environment, such as family members, influenced the adolescent girls’ levels of decision-making autonomy, particularly in-laws. Some sought the comfort of a home delivery so they would have their family members as support, while others were reluctant to deliver in the presence of a male attendant at the hospital. Decisions were mainly made by mothers-in-law, whom the girls relied on information on maternal health. Another qualitative study also observed that women with low decision-making autonomy almost always followed their mothers-in-law’s plans for their pregnancy and delivery care needs [36]. An ethnographic study found that men rarely participate in decisions about maternal health services, corroborating our study findings that mothers-in-law are considered the most experienced person concerning maternal health issues [37]. In line with the findings of other studies conducted in Nepal, our study also showed the importance of mothers-in-law’s decisions for the use of skilled maternal health services and that their knowledge and experience do affect the maternal health-seeking behaviour of adolescent mothers [36,38]. In addition, only a few girls mentioned the role of their husbands in this regard. Some studies conducted in Nepal also suggested that wealth, husband’s education and good communication with the spouse increased antenatal care and delivery-service use [21,39].

Services and efforts of community health workers, neighbours, and women’s group were found to be influential at the community level. The role of CHWs in improving maternal and reproductive health was evident in several developing countries [40, 41]. Regular contract and visits by CHWs help to build trust towards health services and providers. In addition, knowledge sharing platforms, such as “adolescent clubs”, can be productive places for girls to learn about maternal and reproductive health. Several countries have created similar types of committees or organizations in which members of the committee regularly meet to discuss issues related to maternal and reproductive health, including pregnancy, delivery care and contraceptive use [42–44]. A cross-cultural study in Bangladesh and Uganda showed that birth-preparedness and awareness-raising programmes help pregnant mothers seek and demand skilled maternal health services [45].

At the health system level, we found that government programmes and initiatives, quality of care, along with health care provider attitudes, influenced maternal health-seeking behaviour. For example, some adolescent mothers sought maternal health services because of the Safe Motherhood Programme, which provides cash incentives for the use of maternal health services, including ANC, facility delivery, and PNC. Unfortunately, difficulties in accessing these public health facilities, especially during the rainy season, was a barrier for the girls seeking these services, and travel costs were not covered by government incentives. Additionally, girls were deterred by inaccessible health facilities, fixed opening hours, the negative and abrasive attitudes of healthcare providers, along with poor quality of services. Similar studies conducted in Bangladesh showed that shortage of staff and resources, poor infrastructure and the unavailability of trained medical personnel impeded women who were seeking skilled maternal health services [46,47].

Results of this study highlighted several factors which influenced maternal health care-seeking behaviour of married adolescent girls in Nepal. Although this study did not compare the utilization of maternal health services among adolescents and non-adolescent girls, several other studies conducted in Nepal and other developing countries showed that health-care seeking behaviour varies among adolescent and adult women [6, 22, 39, 48]. Being older age, adult women have higher opportunity for education, financial stability and higher decision-making autonomy which positively influenced the uptake of ANC, hospital delivery and PNC services.

### Strengths and limitations of the study

Application of a prospective approach was the main strength of this study. Collection of data from two phases provided a clear understanding of adolescent girls’ maternal health care-seeking behaviour. Data were collected from a district of Terai region of Nepal so the results may not be applicable other regions of Nepal such as Hills and Mountain regions. However, we believe that the results provided an overall and realistic scenario of Terai regions and described the maternal health care-seeking behaviour of married adolescent girls. Another limitation of this study was the focus only on married adolescent girls. However, we decided to consider only married adolescent girls as pregnancy outside marriage is not common and unaccepted by many societies in Nepal.

## Conclusions

This study revealed that adolescent girls’ perceptions, their lack of knowledge about maternal and reproductive health, low decision-making autonomy, the role of family members (particularly mothers-in-law and husbands) and lack of access to and availability of adolescent-friendly health facilities limited their use of skilled maternal health services.

Maternal health programmes targeting adolescent girls should be designed in a way so that they address the broad range of factors influencing their utilisation as revealed in the SEM. Further, health interventions should include family members (i.e. mothers-in-law) and community and sensitize them about the importance of skilled maternal health services and to overcome certain traditional beliefs. Interventions should address barriers to access health facilities and ensure the availability of adolescent-friendly maternal health services. Moreover, health staff should partner with leaders in the community and education sector to empower adolescent girls by keeping them in school and reduce child marriage.

## Supporting information

S1 Interview Guideline IDI guide. This interview guideline was used for recently married adolescent women (non-pregnant).
(PDF)

S2 Interview Guideline IDI guide. This interview guideline was used for recently married adolescent women (pregnant).
(PDF)

S3 Interview Guideline KII guide. This interview guideline was used for key informant interviews.
(PDF)

S1 Transcript Transcript -01. (PDF)

S2 Transcript Transcript-02. (PDF)

S3 Transcript Transcript-03. (PDF)

S4 Transcript Transcript-04. (PDF)

S1 File Supporting Information—Compressed/ZIP File Archive. Interview guideexamples and transcripts.
(ZIP)

## References

[1] World Health Organization. Adolescent Pregnancy: Fact sheet. Geneva: 2014.

[2] United Nations Population Fund. State of World Population 2013: Motherhood in Childhood. New York: 2013.

[3] United Nations Children’s Fund. Progress and prospects. 2014. 10.1016/j.landurbplan.2012.01.010

[4] World Health Organization. Adolescent pregnancy. Fact sheet N°364. Geneva: 2012.

[5] United Nations Population Fund. Adolescent Pregnancy: A review of the Evidence. New York: 2013.

[6] Shahabuddin A, Nöstlinger C, Delvaux T, Sarker M, Delamou A, Bardaji A, et al. Exploring maternal health care-seeking behavior of married adolescent girls in Bangladesh: A social-ecological approach. PLoS One 2017;12 10.1371/journal.pone.0169109 28095432 PMC5240914

[7] Azevedo WF de, Diniz MB, Fonseca ES, Azevedo LM, Evangelista CB. Complications in adolescent pregnancy: systematic review of the literature. Einstein (Sao Paulo) 2015;13:0 10.1590/S1679-45082015RW3127 26061075 PMC4878642

[8] WHO, UNICEF, World Bank G, et al Trends in Maternal Mortality: 1990 to 2015. 2015;32:1–55. ISBN 978 92 4 150363 1

[9] Chandra-Mouli V, Camacho AV, Michaud P-AP-A. WHO guidelines on preventing early pregnancy and poor reproductive outcomes among adolescents in developing countries. *J Adolesc Health* 2013;52:517–22. 10.1016/j.jadohealth.2013.03.002 23608717

[10] United Nations. Goal 5: Improve maternal health. In: *Millennium Development Goals Report*. 2013 29–38. 10.18356/c3bbab7c-en

[11] Anwar I, Kalim N, Koblinsky M. Quality of obstetric care in public-sector facilities and constraints to implementing emergency obstetric care services: Evidence from high- and low-performing districts of Bangladesh. *J Heal Popul Nutr* 2009;27:139–55. 10.3329/jhpn.v27i2.3327 19489412 PMC2761772

[12] Acharya LB, Cleland J. Maternal and child health services in rural Nepal: does access or quality matter more? *Health Policy Plan* 2000;15:223–9. 10.1093/heapol/15.2.223 10837046

[13] Campbell OM, Graham WJ, group Lancet Maternal Survival Series steering. Strategies for reducing maternal mortality: getting on with what works. *Lancet* 2006;368:1284–99. 10.1016/S0140-6736(06)69381-1 17027735

[14] Ministry of Health and Population (MoHP) Nepal New ERA and ICF International Inc. Nepal Demographic and Health Survey 2016: Key Indicators. Kathmandu, Nepal and Rockville, Maryland, USA: 2017.

[15] Human Rights Watch. Our Time to Sing and Play: Child Marriage in Nepal. 2016.

[16] Ministry of Health and Population (MoHP) Nepal New ERA and ICF International Inc. Nepal Demographic and Health Survey 2011. Kathmandu: 2011.

[17] WHO, UNICEF, World Bank G, et al Trends in Maternal Mortality: 1990 to 2015. 2015;32:1–55. ISBN 978 92 4 150363 1

[18] Gill K, Pande R, Malhotra A. Women deliver for development. *Lancet* 2007;370:1347–57. 10.1016/S0140-6736(07)61577-3 17933650

[19] Shahabuddin ASM, Nöstlinger C, Delvaux T, Sarker M, Bardají A, De Brouwere V, et al What Influences Adolescent Girls’ Decision-Making Regarding Contraceptive Methods Use and Childbearing? A Qualitative Exploratory Study in Rangpur District, Bangladesh. PLoS One 2016;11:e0157664 10.1371/journal.pone.0157664 27336673 PMC4919095

[20] Matsumura M, Gubhaju B. Women’s status, household structure and the utilization of maternal health services in Nepal. *Asia Pacific Popul J* 2001;16:23–44.

[21] Furuta BM, Salway S. Women’s Position Within the Household as a Determinant Of Maternal Health Care Use in Nepal. 2001;:17–27. 10.1363/3201706 16723298

[22] Karkee R, Lee AH, Khanal V. Need factors for utilisation of institutional delivery services in Nepal: an analysis from Nepal Demographic and Health Survey, 2011. *BMJ Open* 2014;4:e004372 10.1136/bmjopen-2013-004372 24650803 PMC3963088

[23] Sharma SR, Poudyal AK, Devkota BM, Singh S. Factors associated with place of delivery in rural Nepal. *BMC Public Health* 2014;14:306 10.1186/1471-2458-14-306 24708511 PMC3977667

[24] Chhetri, G. Health Seeking Behaviour of Pregnant Women in Banke District, Nepal. Norwegian University of Science and Technology, Norway. 2015. https://brage.bibsys.no/xmlui/handle/11250/2448285

[25] Sallis JF, Owen N, Fisher EB. Ecological models of health behavior. *Heal Behav Heal Educ Theory Res Pract* 2008;4:465–82. doi: 465–482

[26] Braun V, Clarke V. Using thematic analysis in psychology. Qual Res Psychol. 2006;3: 77–101

[27] Engel J, Glennie J, Samuels F. NEPAL’S STORY: Understanding improvements in maternal health. 2013.

[28] Wagle RR, Sabroe S, Nielsen BB. Socioeconomic and physical distance to the maternity hospital as predictors for place of delivery: an observation study from Nepal. *BMC Pregnancy Childbirth* 2004;4:8 10.1186/1471-2393-4-8 15154970 PMC425583

[29] Tuladhar H, Khanal R, Kayastha S, Shrestha P, Giri A. Complications of home delivery: our experience at Nepal Medical College Teaching Hospital. *Nepal Med Coll J* 2009;11:164–9. 20334062

[30] Pandey S, Lama G, Lee H. Effect of women’s empowerment on their utilization of health services: A case of Nepal. Int Soc Work 2012;55:554–73. 10.1177/0020872811408575

[31] Nour NM. Child marriage: a silent health and human rights issue. *Rev Obs Gynecol* 2009;2:51–6. 10.3909/riog0109 PMC2672998 19399295

[32] Manuscript A, Nanostructures SPC. NIH Public Access. *Nano* 2008;6:2166–71. 10.1021/nl061786n.Core-Shell

[33] Dyson T, Moore M. On Kinship Structure, Female Autonomy, and Demographic Behavior in India Source: Population and Development Review, 9(1) Mar., 1983), pp. 35–60 Published by: Population Council Stable URL: http. 1983;9:35–60.

[34] Basu A, Koolwal G. Two concepts of female empowerment–Some leads from DHS data on women’s status and reproductive health. A Focus gender–Collected Pap Gend using DHS data 2005;:15–33.

[35] Haque SE, Rahman M, Mostofa MG, Zahan MS. Reproductive Health Care Utilization among Young Mothers in Bangladesh: Does Autonomy Matter? Women’s Heal Issues 2012;22 10.1016/j.whi.2011.08.004 21968029

[36] Porter M. The role of mothers–in–law in antenatal care decision–making in Nepal: A qualitative study. The role of mothers-in-law in antenatal care decision-making in Nepal: a qualitative study. Published Online First: 2010 10.1186/1471-2393-10-34 20594340 PMC2910658

[37] Brunson J. Confronting maternal mortality, controlling birth in Nepal: The gendered politics of receiving biomedical care at birth. *Soc Sci Med* 2010;71:1719–27. 10.1016/j.socscimed.2010.06.013 20713304

[38] Shrestha SK, Banu B, Khanom K, Ali L, Thapa N, Stray-Pedersen B, et al Changing trends on the place of delivery: why do Nepali women give birth at home? *Reprod Health* 2012;9:25 10.1186/1742-4755-9-25 23050689 PMC3538619

[39] Dhakal S, Chapman GN, Simkhada PP, van Teijlingen ER, Stephens J, E Raja A. Utilisation of postnatal care among rural women in Nepal. *BMC Pregnancy Childbirth* 2007;7:19 10.1186/1471-2393-7-19 17767710 PMC2075509

[40] Braun R, Catalani C, Wimbush J, Israelski D. Community Health Workers and Mobile Technology: A Systematic Review of the Literature. PLoS One 2013;8 10.1371/journal.pone.0065772 23776544 PMC3680423

[41] Haver J, Brieger W, Zoungrana J, Ansari N, Kagoma J. Experiences engaging community health workers to provide maternal and newborn health services: Implementation of four programs. *Int J Gynecol Obstet* 2015;130:S32–9. 10.1016/j.ijgo.2015.03.006 26115855

[42] Zambon A, Morgan A, Vereecken C, Colombini S, Boyce W, Mazur J, et al The contribution of club participation to adolescent health: evidence from six countries. *J Epidemiol Community Heal* 2010;64:89–95. 10.1136/jech.2009.088443 20007634

[43] WHO. Making Health Services Adolescent Friendly—Developing National Quality Standards for Adolescent Friendly Health Services. World Heal Organ 2012;:3.http://www.who.int/iris/bitstream/10665/75217/1/9789241503594_eng.pdf?ua=1

[44] Schaffer MA, Jost R, Pederson BJ, Lair M. Pregnancy-free club: A strategy to prevent repeat adolescent pregnancy. *Public Health Nurs* 2008;25:304–11. 10.1111/j.1525-1446.2008.00710.x 18666935

[45] Parkhurst JO, Rahman SA, Ssengooba F. Overcoming access barriers for facility-based delivery in low-income settings: Insights from Bangladesh and Uganda. J Heal Popul Nutr 2006;24:438–45. PMC3001147 17591340

[46] Anwar I, Kalim N, Koblinsky M. Quality of obstetric care in public-sector facilities and constraints to implementing emergency obstetric care services: evidence from high-and low-performing. *J Heal Popul* *…* 2009;27:139–55. 10.3329/jhpn.v27i2.3327 19489412 PMC2761772

[47] Islam F, Rahman A, Halim A, Eriksson C, Rahman F, Dalal K. Perceptions of health care providers and patients on quality of care in maternal and neonatal health in fourteen Bangladesh government healthcare facilities: a mixed-method study. *BMC Health Serv Res* 2015;15:237 10.1186/s12913-015-0918-9 26084893 PMC4472247

[48] Haque MN. Individual’s Characteristics Affecting Maternal Health Services Utilization: Married Adolescents And Their Use Of Maternal Health Services In Bangladesh. Internet J Heal. 2009;8

