# Supplementary material for: Maternal health care-seeking behaviour of married adolescent girls: A prospective qualitative study in Banke District, Nepal
Source: PLoS One. 2019 Jun 25;14(6):e0217968. doi: 10.1371/journal.pone.0217968 (PMC6592531; doi:10.1371/journal.pone.0217968)
Supplement: S1 Interview Guideline — This interview guideline was used for recently married adolescent women (non-pregnant). (PDF) [file pone.0217968.s001.pdf]

Research topic: Exploring the maternal and new-born health care-seeking behavior among adolescent women

Target participants:

- Recently married adolescent women (not pregnant)

Type of respondent: Recently married adolescent women<br>Date of interview: __/__/____<br>Place of interview:……………………<br>Time interview started: _______________<br>Time interviewed completed:…………….<br>Name of the interviewer:…………………<br>Code: ………………………..

Topic Guide and questions for In-depth Interview (for recently married adolescent women):

General information:

- Age:
- Education:
- When did you get married (age at marriage):
- Sources of family income:
- Education of the respondent:
- Occupation of the respondent:
- Education of the respondent's husband:
- Occupation of the respondent's husband:

Information about marriage and family planning:

1. How your marriage was arranged? When?
2. Planning to have any child?
3. How do you feel when you think to have a baby?
4. Did you use any family methods after marriage?
   If yes, what types of methods you used?

Knowledge, beliefs and attitude about ANC:

1. What do you think about the importance of care of a woman during pregnancy?
2. Where do women usually go for check-up during pregnancy in this area? And why?
   Probe: about young women
   Probe: In this area, how do women prepare themselves before delivery?
3. What could be reasons why some girls and young women do not go for check-up during pregnancy? (relate back to question 2)
4. To whom you intend to go for your check-up during pregnancy? Why?

Knowledge, beliefs and attitude about Skilled birth attendance and institutional delivery:

1. Where do a woman usually go for child delivery in this village? And why?
2. Where do you prefer to give birth? And why?
3. Do you think, birth at institutional /hospital/medically equipped facility is important? If yes, why? If no, why not?
4. In your opinion why a woman does not go to hospital for child birth in this area?
5. What support do you expect from your family member (husband , parents or parents in law) during pregnancy and delivery?
6. Who takes decision in your family for choosing birth attendant and place of delivery? And why?

Knowledge, beliefs and attitude about PNC:

1. Do you think it is important to check-up a woman and new-born after delivery? If yes, why? If not, why not?
2. What are the common practices in this village of health care seeking behavior of a woman after delivery? Example?
3. According to you, who is the best person to go for check –up after delivery? Why?
4. To whom you would like to go for your check-up after delivery? Why?

Availability and accessibility of skilled maternal and new-born care (ANC, skilled birth attendance, institutional delivery and PNC) services:

1. Where do you go when you have any health problem? Why?
2. How far is the nearest health facility (public or private) for maternal and child health care?
3. What are the difficulties to access the nearest health facility?
4. What are the barriers to access skilled maternal and new-born care of a women in this village? (probe: Are access to health care facility or availability or quality of health care providers are the issues?)

Women's autonomy:

1. How the decision regarding health care service is taken in your family?
2. How you do feel about your position (in terms of decision making) in your family?
3. How do you negotiate about your needs of health care?
4. Are you able to choose the option to go for skilled maternal and child health care (use of ANC from qualified providers, use of skilled birth attendant for delivery, use of PNC institutional delivery) services for you and your child? If yes, why? If not, why not?
