# Supplementary material for: Maternal health care-seeking behaviour of married adolescent girls: A prospective qualitative study in Banke District, Nepal
Source: PLoS One. 2019 Jun 25;14(6):e0217968. doi: 10.1371/journal.pone.0217968 (PMC6592531; doi:10.1371/journal.pone.0217968)
Supplement: S3 Interview Guideline — This interview guideline was used for key informant interviews. (PDF) [file pone.0217968.s003.pdf]

Topic Guide For Key Informant Interview (KII)

Research topic: Exploring the maternal and new-born health care-seeking behavior among adolescent women

Target participant: Policy makers/health care providers

Date of KII: __/__/____<br>Place of KII:……………………<br>Time KII started: ______________<br>Time KII completed:<br>Name of the interviewer…………………………………..<br>Code: ……………………….

General Information:

Designation:
- Duration of work in the current position:
- Major responsibilities:

Questions:

1. How do you see the issue of teenage pregnancy in Nepal? Is it prevalent ? If yes, what are the reasons for that?
2. What are the problems related to it?
3. What are common practices for ANC, delivery care and PNC in Nepal and particularly in this area?
4. Are there differences between adult and adolescent women?
5. What are the potential barriers for a adolescent woman to access skilled maternal (use of ANC, institutional delivery, use of PNC) and new-born health care-services in the context of Nepal?
6. How can culture, social customs or society influence health care-seeking behavior? An example from the Nepal experience?
7. How do you see the issues of women's "autonomy" (i.e. can women come up for their own decisions) in order to access skilled maternal and new-born health care services by a teenage women? What is the scenario in Nepal?
8. What can be done to increase the autonomy (definition and component) of a woman?
9. What are ongoing projects/programs related to maternal and child health in Nepal and what are their activities?
10. Recommendation to improve the health of teenage pregnant women and their new-born?

Note:

- definition of autonomy and components
- definition of skilled maternal and new-born health care
