# Supplementary material for: Maternal health care-seeking behaviour of married adolescent girls: A prospective qualitative study in Banke District, Nepal
Source: PLoS One. 2019 Jun 25;14(6):e0217968. doi: 10.1371/journal.pone.0217968 (PMC6592531; doi:10.1371/journal.pone.0217968)
Supplement: S1 Transcript — (PDF) [file pone.0217968.s004.pdf]

I, Kopila, Echchha and Shahab were late for some reason, so, we reached the Nawbastra health post only around 12 pm by motor vehicle. As we reached Jhuriya, we saw a sign written in red that read, 'Nawbastra Health Post' and just next to it, there was Illaka Police Station. We went inside the health post where Mr. Man Bahadur was attending a patient. The four of us then waited for him to finish checking up the patient. Mr. Man Bahadur came to us and started talking. Just as we were starting the conversation, a sister arrived and gave us her introduction. Her name was Asha Giri who was working as an ANM (Associate Nurse Midwife) at the Nawbastra health post. She also told us that she already knew we were coming to the health post, that's why the staffs there had called upon adolescent pregnant girls there. Since only two of the adolescents met our criteria, Kopila and Echchha took their interview. I on the other hand went inside the ANC (Antenatal Care) room to get the information from the register. The ANC room had 1 bed, 1 table and 2 chairs. With the help from Ms. Asha, we were able to locate adolescent pregnant girls from the ANC register. We decided to go visit them at their homes. We also discussed about the situation and causes of teenage pregnancy at Nawbastra with ANM Asha. She informed us that there was 7-8 percentage of teenage pregnancy among total pregnancy. After that, we called FCHV (Female Community Health Volunteer) Usha on the phone. FCHV Usha said that she will help us gather adolescent pregnant girls at the VDC (Village Development Committee) building. We left for VDC building and after we reached there, we started the interview.

Type of the respondent: Pregnant adolescent girl (Married)

Identification of the respondent: N. 01

Age of the respondent: 17

Address of the respondent: Bankhet-6 Nawabastra

Duration of pregnancy: 5 months running

Name of the respondent's husband: Khojlal Tharu (Raju)

Date of interview: 1st September 2014, 2071/5/16

Place of interview: VDC's Office ground, Nawabastra, Banke, Nepal

Name of the interviewer: Sharmila Regmi

Time interview started: 14:00 PM

Time interview completed: 14:36 PM

Interviewer: Let us start now sister. How old are you?

Respondent: I am 17 years old.

Interviewer: What is your education level?

Respondent: I have studied till 7th grade.

Interviewer: Did you not study further?

Respondent: No!

Interviewer: Why not?

Respondent: Because I got married.

Interviewer: Don’t you want to continue your study?

Respondent: I do want to study further but once you are married you cannot study anymore.

Interviewer: How did you get married?

Respondent: I eloped with my husband.

Interviewer: What does your husband do?

Respondent: He works.

Interviewer: What kind of work?

Respondent: He has gone abroad (India) to work. But I have no idea what kind of work.

Interviewer: How old is your husband?

Respondent: He is 17-18 years old.

Interviewer: What is his education level?

Respondent: He has also studied till grade 7.

Interviewer: So, you two studied together?

Respondent: Yes.

Interviewer: How long have you been married?

Respondent: It has been a year.

Interviewer: Okay, a year?

Respondent: Yes, a year and I am 5 months pregnant now.

Interviewer: Did you use any method of family planning before you got pregnant?

Respondent: No.

Interviewer: Do you know anything about the methods of family planning?

Respondent: No, I don’t.

Interviewer: You do not have any idea about the methods of family planning?

Respondent: No.

Interviewer: After you got married, didn’t you use any method of family planning?

Respondent: No, I didn’t use any.

Interviewer: How did you start to think of having a baby?

Respondent: I don’t really know.

Interviewer: Was it your decision or was it your husband's?

(FCHV arrived)

FCHV: Did you lose your umbrella sister?

Respondent: No.

(Interview continued..)

Interviewer: So the decision of having a baby, was it yours, or was it your husband's?

Respondent: It was a decision made by both of us.

Interviewer: You are still very young; did you face any difficulty during your sexual intercourse?

Respondent: No.

Interviewer: So how did you find out about your pregnancy?

Respondent: I came to know about it when my monthly menstrual cycle stopped.

Interviewer: Menstrual cycle could have stopped due to other reasons too. How were you sure about your pregnancy?

Respondent: I used to feel sick and nauseated. I felt like puking quite often. So, I went for a checkup.

Interviewer: Where did you go for the checkup?

Respondent: There, nearby.

Interviewer: Where?

Respondent: I went to FCHV for the checkup.

Interviewer: How did she check it?

Respondent: She did my urine test.

Interviewer: So, you got the urine tested and came to know about your pregnancy?

Respondent: Yes.

Interviewer: How did you feel when you came to know that you are going to have a baby?

Respondent: I felt really good.

Interviewer: Define good. Are you feeling shy because FCHV is listening to this conversation?

FCHV: Are you feeling shy? Should I leave?

Respondent: No.

Interviewer (To FCHV): Could you sit a little farther from here for now? I think she is feeling shy.

FCHV: Okay, I am going there for now.

Interviewer: Yes please. So tell me, how it feels to know you are going to be a mother?

Respondent: I feel good.

Interviewer: And what does good mean?

Respondent: I don’t know how to explain what it means.

Interviewer: When did you tell your husband that you were going to have a baby?

Respondent: I told him about it as soon as I came to know that I was pregnant.

Interviewer: What did he tell you after he heard the news?

Respondent: He didn’t say anything.

Interviewer: He didn’t say anything?

Respondent: No, he didn’t say anything.

Interviewer: Is this your first child?

Respondent: Yes.

Interviewer: Before this, have you ever gotten pregnant?

Respondent: No.

Interviewer: You are going to be a mother for the very first time, how do you feel?

Respondent: What do I say?

Interviewer: Just tell me how you are feeling?

Respondent: I don’t really know.

Interviewer: Tell me, are you happy or sad or scared? How do you feel?

Respondent: I feel happy.

Interviewer: You are going to have a baby soon, what do you know about the antenatal checkup?

Respondent: I do not know about it.

Interviewer: Have you gone for antenatal checkup?

Respondent: I had gone for checkup once but they did not do my check up.

Interviewer: Why not?

Respondent: I don't know.

Interviewer: What did they tell you?

Respondent: I had gone there when I was 4 months pregnant. But they told me we cannot really know what is happening at 4 months, so they have told me to come only when I am 5 months pregnant.

Interviewer: You are 5 months pregnant now. So, did you go for the checkup?

Respondent: No. Actually they have called me on the 20th of this month (i.e. 5th September). I will go there on the 20th.

Interviewer: Will you go on the 20th (5th September)?

Respondent: Yes, I will.

Interviewer: Why do you think you should go for the checkup?

Respondent: It is because they will check if the baby is safe and sound or not that's why. If I go for the checkup, I can also know if I am healthy or not.

Interviewer: Do the other pregnant mothers in the village also go for check up?

Respondent: Yes, they go for the checkup.

Interviewer: Where do they go for checkup?

Respondent: They go to the health post here at Nawbastra.

Interviewer: Why do you think they go for the checkup?

Respondent: I think they go to make sure that they and their baby are safe and healthy.

Interviewer: Do you think pregnant woman should go for the checkup?

Respondent: Yes.

Interviewer: Where have you decided to go for the checkup?

Respondent: We always go to the health post here.

Interviewer: Do you want to go somewhere else for the checkup?

Respondent: No, I don't want to.

Interviewer: Why do want to go to health post for checkup?

Respondent: It's because the sisters ask us to come to the health post for checkup. They call us there. It is also our health post so better to go there than go somewhere else.

Interviewer: Why is it better to go there than somewhere else?

Respondent: The health post is located in our own VDC and the sisters there are like our own. We know them. That’s why.

Interviewer: You said the health post is better than somewhere else. Do you know anything else about the health post?

Interviewer: The women in this village who are pregnant usually go for the checkup. There isn’t anyone who doesn’t go for checkup isn’t it?

Respondent: Yes, there isn’t anyone as such.

Interviewer: In this village, once the women are pregnant what do they usually do to themselves?

Respondents: I don't know. I have not seen as the pregnant woman who lives near my house hasn't given birth yet.

Interviewer: So you have decided to do the checkup at the health post. Where have you thought of delivering the baby?

Respondent: My thought in it doesn’t matter as wherever my family members take me, I will go there and give birth to my child.

Interviewer: Where do the women in this village usually go?

Respondent: For child delivery?

Interviewer: Yes.

Respondent: Some of them go to *Dus Bigha* (Kohalpur Medical College), some go to Bheri Zonal Hospital.

Interviewer: Where do you want to go to give birth to your child?

Respondent: I don’t really know. The doctor at the health post had referred me to Bheri hospital but since my family members did not give me any money to go there, I didn’t go.

Interviewer: You husband earns isn’t it? Don’t you get to use your husband's money?

Respondent: No, I cannot use it.

Interviewer: Why not?

Respondent: My mother-in-law uses it.

Interviewer: You really wanted to go to Bheri Hospital isn't it?

Respondent: Yes.

Interviewer: Your mother-in-law didn't let you go there. How do you feel when you know you cannot go there?

Respondent: I feel very bad. If I go to Bheri hospital for check up, I can find out about the situation of my baby as well as myself. Now that I know I cannot go, I don't know what to do.

Interviewer: Yes, please express how you truly feel okay?

Respondent: Okay.

Interviewer: At your home, who decides where you should go for checkup and where you should go for delivery?

Respondent: My mother-in-law decides that.

Interviewer: And whatever your mother in law tells you, you do as you are told?

Respondent: Yes.

Interviewer: Do they also consider the decisions you make?

Respondents: Yes, they do.

Interviewer: Then why didn't they let you go to the Bheri Hospital?

Respondents: Maybe they didn't have money that's why they didn't let me go.

Interviewer: Do they let you go to the health post for check up?

Respondent: Yes.

Interviewer: Why did the health workers at the health post suggest you to go to the Bheri hospital?

Respondent: They wanted me to go there so that I can have a proper checkup and find out if I and my child are healthy or not. It would also help me know about the diseases (if I have any). That's why.

Interviewer: Isn't there a facility of blood and urine test at the health post?

Respondent: No.

Interviewer: So they sent you to Bheri Hospital for blood and urine test?

Respondent: Yes.

Interviewer: Have you ever tested your blood and urine?

Respondent: No, I haven’t.

Interviewer: So you have not done any test except the pregnancy test?

Respondent: Yes, I haven’t done any.

Interviewer: Do you want to have your blood and urine tested?

Respondent: Yes. I will go to test my blood and urine once my sister arrives.

Interviewer: Which sister?

Respondent: My own sister.

Interviewer: Why only when she arrives?

Respondent: Because she can take me to the hospital and get my blood and urine tested. Also, when I ask her for money, she will give it to me. My sister stays far from here. She is in Kathmandu right now. That’s why I will go after she gets here.

Interviewer: Wont your mother-in-law take you there?

Respondent: No.

Interviewer: If your husband was here, would he take you to the hospital?

Respondent: Yes. Definitely!

Interviewer: Where do you want to give birth to your child?

Respondent: Wherever it's easy for me to give birth. If I had given birth to a child before, I would have known. So, I don’t know for sure.

Interviewer: But still, you might have thought about it. What sort of place might be good for you and your child?

Respondent: I think the health post will be appropriate for delivery. I don’t really know where else to go.

Interviewer: Don’t you have any wish as such about where you want to give birth?

Respondent: I think I will give birth to my child at the health post.

Interviewer: Which health post? Is it the one which is very near form here?

Respondent: Yes.

Interviewer: Why do you think health post will be appropriate choice for the birth of your child?

Respondent: Because they take care of us properly at the health post. That’s why.

Interviewer: What do they do?

Respondent: They are very concerned about us. They care for us. I hear they don’t care about the patient at all at *Dus Bigha* (Kohalpur Medical College) so I am scared to go there.

Interviewer: From whom did you hear that?

Respondent: From friends and sisters.

Interviewer: Why did they tell you *Dus Bigha* isn’t good?

Respondent: I heard the health workers there don’t look after the patients properly.

Interviewer: Do they look after you properly at the health post?

Respondent: Yes, they do. But if I won't be able to give birth to my child at the health post, I will have to go to *Dus Bigha*.

Interviewer: In what condition do you think you might be not be able to deliver your baby at the health post?

Respondent: I don’t really know but I heard one cannot give birth at a very small age.

Interviewer: Why do you think one cannot give birth at small age?

Respondent: I don’t know.

Interviewer: Why do you think very young aged women cannot give birth while older women can?

Respondent: Maybe it is because small aged women aren’t mature enough to give birth to a child whereas the older women are already matured and are able to give birth easily.

Interviewer: Mature in what sense?

Respondent: Maybe their body is already properly matured. I am not too sure.

Interviewer: So you will go for the checkup soon. Wont you?

Respondent: Yes, I will.

Interviewer: You will be going on the 20th (5th September) right?

Respondent: Yes.

Interviewer: Who do you wish would do your checkup?

Respondent: I wish a female doctor would do my check up.

Interviewer: Are there female doctors available?

Respondent: Yes.

Interviewer: You are going for the checkup during your prenatal period i.e. before delivery. Do you think it is necessary for you to go for the checkup even after the delivery of your child?

Respondent: Yes I think it is necessary.

Interviewer: Why do you think it's necessary?

Respondent: I don’t know why.

Interviewer: You just said it is necessary to go for checkup even after the delivery. Why do you think so?

Respondent: ……. (Pause, Puzzled)

Interviewer: Checkup is necessary during the pregnancy as well as after the delivery of the child. Why do you think so? Can you tell me?

Respondent: I think it is necessary after the delivery to find out if the body of the mother is healthy or not.

Interviewer: Yes, that is good. Tell me more.

Respondent: I don’t know anymore.

Interviewer: After you give the birth to your child, where have you thought of going to for checkup?

Respondent: I don’t know if they do the checkup at the health post or not. So I will be going to Bheri Zonal Hospital.

Interviewer: You will go to Bheri Hospital?

Respondent: Yes.

Interviewer: Isn't there a provision of checkup at the health post after delivery?

Respondent: I don’t know.

Interviewer: Why do you want to go to Bheri Hospital?

Respondent: Because the health post also usually sends us there. That’s why.

Interviewer: By whom do you wish to get checked up at the health center?

Respondent: What do you mean? I didn’t get you.

Interviewer: Okay. Let me give you an example. Do you prefer doctors or nurses or FCHV for doing your checkup?

Respondent: I don't think FCHV will do the checkup. Maybe nurses and doctors, big doctors.

Interviewer: Well all of them are responsible for checking up the patient, but I am asking, whom do you prefer in specific when you are at the health center?

Respondent: I don't know whom I should choose. I wish to be checked up by the nurse because with doctors, I feel a little uncomfortable to talk to. Whereas I can easily talk to nurses about my problems.

Interviewer: Yes, you should openly express your viewpoint like this. That way, things will happen the way you want them to happen.

Interviewer: Whenever you have any health problems, where do you usually go for treatment?

Respondent: There is only one health post around here. Where would I go except there?

Interviewer: Where have you been going till date?

Respondent: Only to the health post.

Interviewer: How far is the health post from your home?

Respondent: It's near from here.

Interviewer: How near? What is the distance? How much time does it take you to reach there?

Respondent: I don't know, I do not look at the time.

Interviewer: No, but you might know how much time does it takes approximately? 15-20 minutes, half n' hour, how much?

Respondent: I think 15-20 minutes approximately.

Interviewer: How do you get to the health post?

Respondent: I walk to the health post.

Interviewer: Do you have to face any difficulty while going to the health post?

Respondent: No.

Interviewer: Do your family members let you visit the health post?

Respondent: Yes.

Interviewer: Why? Is it because the health services are free of cost at the health post? Why do your family members let to go to the health post but not to Bheri Hospital?

Respondent: Actually they have told me I can go to Bheri hospital if I have the money required to go there. But I don't have the money to go to Bheri hospital.

Interviewer: What about the health post?

Respondent: Well it's near and also I need to visit it quite often. So they let me go to the health post.

Interviewer: At your home, who tells you to go for antenatal check up and who decides which hospital you will be taken to?

Respondent: FCHV tells me to go for antenatal check up. And about taking me to the hospital, I don't know who tells it. I just go on my own.

Interviewer: You go on your own. Doesn't your mother-in-law stop you?

Respondent: No

Interviewer: You mentioned earlier that you want to go to Bheri hospital and you feel very bad that you couldn't go because don't have money. If you had enough money to go there, would you go to the hospital?

Respondent: Yes.

Interviewer: When your husband sends the money from abroad (India), won't you keep it?

Respondent: No, my mother-in-law will not give it to me.

Interviewer: How will you tell your mother-in-law if you will want to go to Bheri hospital?

Respondent: I have already told her, I will not tell her again.

Interviewer: Why not? You will have to go for the sake of your child.

Respondent: I already told her about this once. How many times should I tell her now? She has already told me she doesn't have any money. What should I do?

Interviewer: You should tell her again.

Respondent: No, I won't.

Interviewer: If your husband was here with you, would you tell him?

Respondent: When my husband was here, he didn't go to health post with me.

Interviewer: In your community, what sort of practice is followed once a mother delivers her child?

Respondent: I don't know.

Interviewer: No, you might know that after you give birth to your baby, there must be some kinds of tradition and culture in your family that are being followed?

Respondent: I don't know.

Interviewer: Okay, I will give you an example. For instance, the family members and relatives provide nutritious food to the mother and new clothes to the new born child as well as the mother. There is also a practice of body massage that is given to both mother and her child. Does your family member practice these kinds of things?

Respondent: Yes, they do.

Interviewer: What are the things they do to the new born child and the mother?

Respondent: Yes, some people from my village give body massage to the mother and new born child. But I don't know about the practice of providing new clothes to them.

Interviewer: Don't they give you adequate nutritious food to eat during this time? More than the usual inorder to meet your nutritional requirement?

Respondent: I don't know.

Interviewer: At present, you are 5 months pregnant. What kind of things do you wish your family members did during this time to support you?

Respondents: What kind of things?

Interviewer: Yes. What kind of things? Like the one you mentioned earlier, you wished they gave you some money isn't it?

Respondent: (Takes long breath)

Interviewer: Are you okay? Do you feel uncomfortable?

Respondent: No. I don't know how to say.

Interviewer: Please tell me exactly how you feel, how you wish your family supported you. Don't hesitate. I am not going to tell anyone.

Respondent: …. (Pause) I wish they supported me in my chores. I don't know what else to say.

Interviewer: Do you also wish they would take you to the health post?

Respondent: No, health post is nearby.

Interviewer Then, do you want them to let you go to Bheri hospital?

Respondent: Yes.

Interviewer: Do you have heavy household workload?

Respondent: Yes, we have so much work to do.

Interviewer: Okay. That's why you have come to the mill here to do your chores, isn't it?

Respondent: Yes.

Interviewer: Do you face difficulty while doing your work?

Respondent: Yes.

Interviewer: Why do you wish they supported you in your work?

Respondent: It is very difficult to work alone, especially when I have to carry heavy objects. If there were one to two more people to support me, the work would be easier.

Interviewer: Do you have any problem while going for the antenatal checkup?

Respondent: No.

Interviewer: Is there any problem while going for the child delivery?

Respondent: No, there isn't.

Interviewer: Is there a problem while going for the post-natal checkup?

Respondent: I will know about this once I give birth to my child. (Laughs!)

Interviewer: Where do you want to give birth to your child?

Respondent: I don't know where I should say.

Interviewer: Tell me where you want to have your delivery at home, at the health post, at *Dus Biga* (Kohalpur Medical Colege) or at Bheri hospital?

Respondent: I want to have my delivery at the health post.

Interviewer: Why at the health post and not Bheri hospital?

Respondent: Because health post is near from here.

Interviewer: So most of the women in this village deliver their baby at the health post is it?

Respondent: Yes.

Interviewer: Do you have any idea about the problems women face during the delivery?

Respondent: …. (Pause)

Interviewer: Do you think there might be a problem during child delivery?

Respondent: I don't know.

Interviewer: What about during pregnancy? Could there be any problems during pregnancy?

Respondent: I don’t really know about this.

Interviewer: You mentioned earlier that small/young aged women have difficulty in giving birth whereas mature women don’t have to face such difficulty.

Respondent: Yes, I did.

Interviewer: Why do you think there is difference between the child delivery process of small aged women and mature women?

Respondent: I heard people say it's because of the age.

Interviewer: What do they say?

Respondent: They say, if a girl gets married at a very small age, she has to face difficulty while giving birth to her child. So, a girl should marry when she is older.

Interviewer: Difficulty? What kind of difficulty?

Respondent: I don’t know.

Interviewer: You know they have to face difficulty but you don’t know what kind of is it?

Respondent: Yes.

Interviewer: You are going to be a mother soon, have you started preparing for it?

Respondent: No.

Interviewer: Why not?

Respondent: I don’t know.

Interviewer: Tell me about your family. Who are there?

Respondent: My mother-in-law, brother-in-law, sister-in-law and my husband.

Interviewer: What about your father-in-law?

Respondent: No, he isn’t there.

Interviewer: There are a total of 4 members in your family right now. A new member is going to come soon. Have you not started preparing for it?

Respondent: No.

Interviewer: Don’t you think you need to prepare for it?

Respondent: I don’t know. They have not done any preparations for it.

Interviewer: If others won't do, it doesn’t matter. But do you think preparation needs to be done before hand?

Respondent: I don’t know. What kind of preparations should I do?

Interviewer: Preparations like, preparing enough food and storing them properly for its use during emergency, collecting and saving money, preparing stack of clothes and other necessary goods required during and after deliver etc. What do you think of this?

Respondents: After the baby is born, my family, sisters and relatives from maternal home will bring them to me.

Interviewer: Yes, they might bring to you once the baby is born, but shouldn’t you also be preparing for it from right now?

Respondent: Yes.

Respondent: Is he calling you? (Referring to the person nearby)

Interviewer: No, he is our Sir. He has come to check if we are carrying out the interview properly or not. Like I said, we have come here to conduct a study. Do you want to ask any question?

Respondent: No.

Interviewer: If you have any queries, please feel free to ask them.

Respondent: Okay.

Interviewer: Have you worked somewhere before?

Respondent: No.

Interviewer: Didn’t you study after you got married?

Respondent: No, I didn’t. How could I study? Once I got married, I had to perform all household chores.

Interviewer: Did you want to study further?

Respondent: Yes, I wanted to but what to do, I cannot. The family members from my maternal home also wanted me to study further but I have to do the household work, so I can't.

Interviewer: How is your family expenses managed?

Respondent: By performing general work.

Interviewer: You husband has gone to India to earn the living, what else are the income source of your family?

Respondent: We have our own land. We make our living from it.

Interviewer: Is that enough to cover your family expenses? How much is the monthly expenditure of your family?

Respondent: I am not responsible for managing the expenses of the family, so I don’t know.

Interviewer: Do you sometimes think that if you had money, you would do this, do that.

Respondent: Yes, I think about it.

Interviewer: What would you do if you had the money with you?

Respondent: I would utilize it for going to the checkup whenever necessary.

Interviewer: Do you sometimes have the craving for food and things like that; that you think you would buy if you had the money?

Respondent: My maternal home provides me the food.

Interviewer: What else does the mother's side of your family give you?

Respondent: They also give me money. I have been collecting that money.

Interviewer: Are they still giving you money?

Respondent: Yes.

Interviewer: What kind of food do you normally buy and eat from that money?

Respondent: I don’t buy and eat food from that money. I am collecting and saving them. (Laughs)

Interviewer: Why are you saving them?

Respondent: Just like that.

Interviewer: After you got pregnant, have you started intaking the food more that you usually do? Or have you been eating the same amount of food which you normally consumed when you were not pregnant?

Respondent: After I got pregnant, I have been eating the fruits that are sent from my maternal home.

Interviewer: What kinds of fruits?

Respondent: Fruits like Pomegranate, Apples etc.

Interviewer: Do you eat them alone or do you share it with your family members?

Respondent: I usually eat them alone but when I am not at home, I think my family members eat it.

Interviewer: Have you seen them eating your fruits?

Respondent: My sister-in-law is small, so she eats them without telling.

Interviewer: Your sister in law is small?

Respondent: Yes, I have once sister-in-law, she is small.

Interviewer: You are going for the ANC checkup on the 20th (5th September). When will you be going for check up after that?

Respondent: Whenever they will call me after that, I will go.

Interviewer: You will only go after they will call you?

Respondent: Yes.

Interviewer: Why?

Respondent: If I go there without the health workers at the health post calling me, the doctors might be absent. So I will only go when they call me.

Interviewer: Do you know about the health problems during pregnancy?

Respondent: No.

Interviewer: And you haven’t also been preparing for the delivery right?

Respondent: Yes, I haven’t.

Interviewer: Haven't the FCHVs taught you regarding the preparation that needs to be done before delivery?

Respondent: No.

Interviewer: Haven't any sisters of yours told you what needs be done before delivery?

Respondent: No, I have one elder sister but she isn’t married.

Interviewer: Do you want to ask any question?

Respondent: No

Interviewer: At your home, who decides what you will be eating and where you will be giving birth to your child?

Respondent: All these decisions are made by my mother in law.

Interviewer: Do they also consider your viewpoint while making a decision?

Respondent: Yes, they do.

Interviewer: When you realize you are not able to go for the check up because you dont have money, how do you feel?

Respondent: I feel very bad, what to do?

Interviewer: What kind of bad feeling?

Respondent: If I had the money in my hand, I could have gone for the check up anytime and anywhere.

Interviewer: Are you bored? I have been asking repetitive questions.

Respondent: No.

Interviewer: Are you in a hurry? Do you have to go somewhere?

Respondent: No.

Interviewer: You don't talk much. Are you feeling shy because of me?

Respondent: No.

Interviewer: Are u feeling uncomfortable due to this heat?

Respondent: No, there is shade here.

Interviewer: Do you have a problem to go to Bheri?

Respondent: There isn't any problem.

Interviewer: Why do you want to deliver your child at the health post?

Respondent: I have heard people say it's easy to deliver the baby at the health post.

Interviewer: In what sense is it easy?

Respondent: I don't really know.

Interviewer: And what difficulty might be there at Bheri hospital?

Respondent: I don't know about it.

Interviewer: Maybe because you are going to deliver your first child, you don't know much about it.

Respondent: Yes.

Interviewer: Does your mother in law teach you anything about it?

Respondent: No.

Interviewer: Why?

Respondent: They never go to the health center that's why.

Interviewer: Okay. Where do the women in this village usually go to get the health services?

Respondent: To *Dus Bigha.*

Interviewer: Why do they go there?

Respondent: I don't know.

Interviewer: Eventhough there is health post nearby, why do you think women go to *Dus Bigha*?

Respondent: Maybe because their family members take them there.

Interviewer: Why do you think instead of taking to the health post, the family members take them to Dus Bigha?

Respondent: I don't know.

Interviewer: Your decision to give birth to your child at the health post, is it your own decision or is it the decision made by your mother in law?

Respondent: It is my own decision.

Interviewer: Why?

Respondent: I don't know.

Interviewer: Tell me, why you want to give birth at the health post and not at the Bheri hospital?

Respondent: I don't know why.

Interviewer: You don't know or you are feeling shy to talk about it?

Respondent: I am not shy. It's just that I don't know what to say.

Interviewer: So you are not feeling shy?

Respondent: No.

Interviewer: If there is anything you want to ask, please go ahead.

Respondent: (Nodding) No.

Interviewer: During your delivery, what do you wish to have with you?

Respondent: I wish my husband will be there with me during delivery.

Interviewer: In absence of your husband during this crucial time, do you have to face difficulty in your everyday life?

Respondent: There is difficulty, but what to do? I have to work anyway.

Interviewer: If your husband was here with you, why would it be easy for you?

Respondent: If he was here, he would be there for me all the time. Whatever I needed, he would fulfill them.

Interviewer: Would he also support you in your chores if he were here?

Respondent: Yes, he would even support me in my work.

Interviewer: Tell me more about the kinds of problems are you facing in absence of your husband?

Respondent: There isn’t anyone to support in my work. These kinds of problems are there.

Interviewer: With whom do you go to the health post?

Respondent: I go there alone.

Interviewer: If your husband was here, would you go with him?

Respondent: Even if he was here, I would go there alone.

Interviewer: Why so? Wouldn’t he accompany you to the health post?

Respondent: …. (Pause) I feel shy to go to the health post together. (Laughs out loud)

Interviewer: What is there to feel shy about?

Respondent: There aren’t many male people at the health post, only females. That’s why I feel shy to go together.

Interviewer: If that is the case, he could just stay outside when you enter the health post. Do you what to add anything or ask anything?

Respondent: No.

Interviewer: Our interview ends here. Thank you so much for you support.

Conclusion:

After the interview, I concluded that she was aware about the ANC checkup and she preferred institutional delivery. She depended upon her mother in law for money as she cannot use her husband's money. She doesn’t know actual side effects of teenage pregnancy.
