# Supplementary material for: Maternal health care-seeking behaviour of married adolescent girls: A prospective qualitative study in Banke District, Nepal
Source: PLoS One. 2019 Jun 25;14(6):e0217968. doi: 10.1371/journal.pone.0217968 (PMC6592531; doi:10.1371/journal.pone.0217968)
Supplement: S2 Transcript — (PDF) [file pone.0217968.s005.pdf]

We reached Khajura Manakama Chowk at 12:15 PM. FCHV Shova BK informed us that 3 pregnant adolescent girls were there. First of all, FCHV Shova BK took us to the home of a woman. We reached there and explained about the objectives of our study. We then asked the age of the woman. Since she was 24 years old, we left and went to another house. The next house was my respondent's house. When I got there, my respondent's sister in law was preparing tea in her shop. I introduced myself and requested her to call my respondent there so that I could take her interview. After 10-15 minutes of wait, I met my respondent. After introducing myself, I explained about the study objectives and asked for her time. When she agreed to give the interview, I requested them to arrange for a place to sit for the two of us. I even talked to the other family members like the respondent's husband and mother in law and explained about the study objectives. After taking the consent from the family members, I started the interview.

Type of the respondent: Pregnant adolescent girl (Married)

Identification of the respondent: 01- Bagyashwari

Age of the respondent: 19 years

Address of the respondent: Bagyashwari -5, Manakamana Chowk - Banke

Duration of pregnancy: 5 months (Expected date of delivery: December)

Date of interview: 3rd September 2014

Place of interview: Respondent's house

Name of the **Interviewer**: Echchha Ku. Pun

Time interview started: 1:05 PM

Time interview completed: 1:55 PM

Interviewer: When did you get married?

Respondent: A year ago.

Interviewer: How did you get married?

Respondent: It was a love marriage.

Interviewer: What is your level of education?

Respondent: Bachelors.

Interviewer: What kind of work are you doing?

Respondent: I don't work. I just stay home.

Interviewer: Did you used to work before?

Respondent: No.

Interviewer: What is your husband's level of education?

Respondent: Bachelors.

Interviewer: What kind of work does your husband do?

Respondent: He doesn't work.

Interviewer: Why? Does he work at home and at your shop?

Respondent: No, there isn't much work to do at home

Interviewer: What are the income sources of your family?

Respondent: My father in law's monthly pension.

Interviewer: Okay. Where did he work?

Respondent: He was in Indian Army.

Interviewer: Any other income sources?

Respondent: My brother in law has also gone to India to work there.

Interviewer: Did you plan beforehand to get pregnant?

Respondent: No, it just happened. (Laughs)

Interviewer: Didn't you use any methods of family planning after you got married?

Respondent: No, we didn't use any.

Interviewer: Why didn't you use it?

Respondent: People say you shouldn't use family panning method until you have had your first child.

Interviewer: Why? What will happen if you use it?

Respondent: I don't know.

Interviewer: There must be some reason behind it, tell me why do you think you should not use family planning method before you have your first child?

Respondent: After the intake the family planning pills, it will have side effects that's why.

Interviewer: What kind of effect? Do you think a woman will not be able to conceive if she takes the pills?

Respondent: Yes.

Interviewer: What do you know about the methods of family planning?

Respondent: I know about oral pills and injection.

Interviewer: Do you know the name of that injection?

Respondent: *Sangini* injection. (Means: Depo-Provera (DMPA) injectable contraceptive)

Interviewer: You are a pregnant woman now. How did you feel when you came to know you were pregnant?

Respondent: I felt good.

Interviewer: How did you confirm your pregnancy?

Respondent: I confirmed about it by checking at home.

Interviewer: What did to check be confirm?

Respondent: I brought the pregnancy test kit and got my urine tested.

Interviewer: After pregnancy, have you visited anyone for the prenatal check up?

Respondent: Yes, I have.

Interviewer: Whom did you visit?

Respondent: I went to visit doctor Usha for check up when I had a stomach ache.

Interviewer: How many times have you gone for the prenatal check up?

Respondent: Twice. 1st to get an injection and 2nd time for the check up.

Interviewer: Where did you go to get these health services? To the health post or to the medical?

Respondent: For getting an injection, I went to government health institution. My husband usually takes me wherever I need to go, so I don't know much.

Interviewer: Where do the women in this village usually go for ANC checkup?

Respondent: I don't know much about it.

Interviewer: Still, you might know a little. Tell me where do they usually go?

Respondent: They either go to the PHCC or to doctor Usha.

Interviewer: So, all the pregnant women go there?

Respondent: Yes, most of the women I know go there.

Interviewer: Is there anyone who doesn't go?

Respondent: No, all of them go.

Interviewer: In this community, how do the women prepare themselves for the child delivery, once they get pregnant?

Respondent: For the delivery of the child?

Interviewer: Yes, what kinds of preparation do they start doing after they get pregnant?

Respondent: They start collecting and gathering the clothes the upcoming baby will need, they also arrange the goods and materials the mother needs during and after child birth.

Interviewer: Do they also prepare for an arrangement of a blood donor which a mother might need incase of an emergency situation?

Respondent: I don't know about that.

Interviewer: In your opinion, how necessary is proper care and support to a woman during pregnancy?

Respondent: I think it is very necessary.

Interviewer: Why do you think it's necessary?

Respondent: When we visit the hospital, we can get every details and information which are essential during pregnancy. So it will be better for us.

Interviewer: Better? How?

Respondent: Better because we will have the essential information of all the things that we need to know during pregnancy. It will be better for us as well as our children soon to be born.

Interviewer: Why did you visit Dr. Usha for your prenatal checkup?

Respondent: I had a pain in my lower abdomen. That's why I visited her.

Interviewer: What did she tell you then?

Respondent: She told me there wasn't adequate blood in my body. (Means: Anaemia)

Interviewer: Did you do the blood test?

Respondent: Yes.

Interviewer: So, did she give you any medication?

Respondent: Yes, she gave me medicine which I need to take for a month. She told me it will increase my blood level.

Interviewer: Do you know about the reasons behind women not going for ANC check up in this community?

Respondent: I don’t know about it as most of the women I know go or ANC check up.

Interviewer: Where do the women in this village go for child delivery?

Respondent: They go to the hospital.

Interviewer: Why do they go to the hospital?

Respondent: We don’t have any child delivery facilities at home. But, at the hospital, there are many facilities. The delivery process can go smoothly and it will also be safe for the child. That’s why.

Interviewer: How will it be safe for the child?

Respondent: If the child has any health problems, it can get prompt treatment at the hospital. So, it is better for the child.

Interviewer: And where have you thought of giving birth to the child.

Respondent: At the hospital.

Interviewer: Why have you decided to give birth at the hospital?

Respondent: Any kind of health problems can be treated and solved in time at the hospital, that’s why.

Interviewer: In your opinion, how important is it to have the child delivery at a safe and secure place and by a trained and skilled health professional?

Respondent: It is indeed very important.

Interviewer: Why do you think it’s important?

Respondent: That way, any type of health problems can be taken care of in time and mishaps can be prevented.

Interviewer: Do you know anyone who doesn’t go to the hospital for child delivery?

Respondent: No, there is no one as such.

Interviewer: In this crucial period of pregnancy and delivery, what kind of expectations have you kept from your husband and your family?

Respondent: I expect them to tell me and teach me about the things that I do not know about, which are necessary for me and my child.

Interviewer: Any other expectations?

Respondent: I don’t know any others.

Interviewer: What do you expect from your husband?

Respondent: I wish he would give me all the love and care.

Interviewer: Have you felt the difference between the attitude of your family (Your father-in-law, mother-in-law) towards you before the pregnancy and after pregnancy?

Respondent: Yes, there is a difference.

Interviewer: Is it so?

Respondent: Yes, they love/care for me now.

Interviewer: In your family, who decides by whom you will have your child delivered and where?

Respondent: It is decided by my father in law and mother in law.

Interviewer: Why do they decide? Don't you get to decide in these matters?

Respondent: Yes, but they are the elders, it will be better if they decide. We should respect their decision.

Interviewer: In your view, how important is it for the mother and the new born child to go for the post-natal check up (PNC)?

Respondent: I think it is important.

Interviewer: Why do you think so?

Respondent: It is important to make sure both the mother and child are healthy and there isn't any health problem.

Interviewer: What are the benefits of PNC check up to a new born child?

Respondent: The child can get immunized and his growth also can be monitored, that's why it is important.

Interviewer: And what are the benefits of it to the mother?

Respondent: It can be ensured that the mother is healthy and no negative health effects are there.

Interviewer: After you have delivered your first child, what do you think can be done to maintain the birth spacing between your first child and your second?

Respondent: I don't know.

Interviewer: Who do you think will be appropriate for you to get a check up by after your child delivery?

Respondent: I didn’t get you.

Interviewer: I meant do you think it will be better for you to get a check up by the health workers from government health institution or by a private doctor?

Respondent: I think it will be better if the doctor does the check up.

Interviewer: Why do you think so?

Respondent: Because the big doctors have adequate knowledge about everything, that’s why.

Interviewer: Are all the women in your community using the health services?

Respondent: What kind of health services are you talking about?

Interviewer: I meant, do all the women in your community go for ANC, PNC check up and new born check up?

Respondent: Yes, all of them go for it.

Interviewer: Do you know anyone who doesn’t go?

Respondent: No.

Interviewer: When there is any kind of health related problems in your family, where do you usually go for treatment?

Respondent: We go to the hospital.

Interviewer: Why do you prefer going to the hospital?

Respondent: Because the health problems can be treated properly at the hospital.

Interviewer: Where is the nearest health center from here?

Respondent: I don’t know much, I think the one in ward no. 1 (PHCC) is the nearest from here.

Interviewer: How long does it take to reach the PHCC from here?

Respondent: I think about 10-15 minutes.

Interviewer: Are there any kind of difficulties in reaching the PHC from your home?

Respondent: Yes, it’s a bit difficult as there isn’t availability of any transportation facility to reach there. We have to walk.

Interviewer: Do you have to face any difficulty to go there due to your family members?

Respondent: No.

Interviewer: Is it easy for the women of this community to use the health services like; ANC, PNC, child delivery services etc?

Respondent: Yes.

Interviewer: Who is responsible for deciding about the use of health services in your family?

Respondent: My father in law, mother in law and all of us.

Interviewer: Are you happy with their decision?

Respondent: Yes, I am.

Interviewer: If a situation comes up, where you have to make a decision to fulfill your health needs, will your family members accept your decision/suggestion?

Respondent: Yes, they will accept my decisions too.

Interviewer: Are there any difficulties in accessing maternal and new born health care services? Do you have any problem to get to the health center which provides these services?

Respondent: No, there aren't any problems.

Summary:

After the conversation, I concluded that, my respondent is educated and has some knowledge but not adequate knowledge. Practice of ANC is good because she visited the health center twice.

Memo:

I think the family background of my respondent is very good as she seems very happy. She gets all the love and care from her family.
