# Supplementary material for: Maternal health care-seeking behaviour of married adolescent girls: A prospective qualitative study in Banke District, Nepal
Source: PLoS One. 2019 Jun 25;14(6):e0217968. doi: 10.1371/journal.pone.0217968 (PMC6592531; doi:10.1371/journal.pone.0217968)
Supplement: S3 Transcript — (PDF) [file pone.0217968.s006.pdf]

We reached Naubasta health post at 12:00 PM. The ambiance around the health post was very noisy and crowded. We directly went inside the OPD room where the health post incharge was examining a patient. After waiting for about 5 minutes there, the patient left and we started giving our introduction to the health post incharge. We also explained our objectives for visiting the health post. The health post incharge then informed us that he has already made an arrangement for the respondents required for our study through the FCHVs. We (Kopila & Echchha) entered the Maternal and child health (MCH) room where 4 people were there. We introduced ourselves and again about our objectives for coming there. The nurse at the heath post introduced us to our respondents. I asked them about their duration of pregnancy. One of the woman was 5 months pregnant and the other was 8 months pregnant. I asked the 5 months pregnant women to give me her time for the interview and also kindly requested the 8 months pregnant woman to head back home.

I requested the nurse at the health post to arrange a place where I could smoothly conduct my interview. The nurse then showed us the conference hall where I and my respondents started conversing about the study objectives, phases of the study, its advantages, as well as about the process of the interview. After the discussion, my respondent agreed to give the interview.

Type of the respondent: Pregnant adolescent girl (Married)

Identification of the respondent: 02- Naubasta

Age of the respondent: 17 years old

Address of the respondent: Naubasta - 5, Banke

Duration of pregnancy: 5 months

Date of interview: 1st September 2014, 2071/5/16

Place of interview: Naubasta Health Post

Name of the interviewer: Echchha Ku. Pun

Time interview started: 12:10 PM

Time interview completed: 1:00 PM

Interviewer: How old are you?

Respondent: I am 17 years old.

Interviewer: What I your level of education?

Respondent: I am illiterate.

Interviewer: What do you do for living?

Respondent: I don't do anything, I stay home.

Interviewer: When did you get married?

Respondent: Last year in Ashadh (June-July).

Interviewer: What are your sources of income?

Respondent: My husband is a labourer.

Interviewer: Is your husband's earning enough to cover up your daily expenses?

Respondent: My father in law also a rickshaw driver. We buy our daily requirement of vegetables and spices with that. We manage our expenses this way.

Interviewer: What is your husband's level of education?

Respondent: He has studied till 5th grade.

Interviewer: How did you get married?

Respondent: I eloped with my husband. It was a love marriage.

Interviewer: How long have you been married?

Respondent: It has been a year.

Interviewer: Did you plan beforehand for getting pregnant with your baby?

Respondent: No, it just happened.

Interviewer: After you got married, did you use any method of family planning?

Respondent: Yes.

Interviewer: Which method of family planning did you use?

Respondent: (Pause)

Interviewer: From whom did you take the suggestions to use the method of family planning?

Respondent: From my husband.

Interviewer: Which method of family planning did you use?

Respondent: We didn't use any.

Interviewer: Do you know anything about the methods of family planning?

Respondent: No, I don't.

Interviewer: After you found out that you were pregnant, who was the first person you informed about it?

Respondent: I told my husband first.

Interviewer: What did your husband say then after?

Respondent: He took me to the health post for check up.

Interviewer: After you went to the health post, what did they examine?

Respondent: The nurse got my urine tested and confirmed my pregnancy.

Interviewer: How did you feel when you found out you were pregnant?

Respondent: My monthly menstruation cycle stopped after I became pregnant.

Interviewer: What kind of feeling did you have after you found out you were pregnant?

Respondent: I felt dizzy and also had the urge to vomit at times.

Interviewer: What else happened?

Respondent: I had slight fever as well as headache at that time.

Interviewer: Weren't you happy when you found out you were pregnant?

Respondent: Yes, I was very happy.

Interviewer: Where do the women in your community usually go for the ANC check up?

Respondent: To the health post.

Interviewer: Is there any woman who doesn't go to the health post for ANC check up?

Respondent: No, all of them go there.

Interviewer: Why do you think they go to the health post for check up?

Respondent: Because it will be better for the mother and child. It is easy to have the child delivered at the health post. They also give money to the mother and clothes to the child at the health post. That's why.

Interviewer: Do you have any other reasons for going to the health post?

Respondent: No.

Interviewer: After the women get pregnant, what kinds of preparation do the women usually do for the child delivery?

Respondent: They start gathering and collecting money as well as clothing which are necessary during that time.

Interviewer: What else do they prepare?

Respondent: That is all.

Interviewer: Don't they start preparing and storing nutritious food items?

Respondent: Yes, they do that as well.

Interviewer: When do the women start intaking extra foods (dietary supplements) to meet their dietary requirements?

Respondent: After the delivery, women start taking extra foods.

Interviewer: Aren't the women provided dietary supplements during the pregnancy?

Respondent: Yes, they are provided extra foods.

Interviewer: What kinds of food are they provided?

Respondent: They are provided fruits like; banana, apple, pomegranate etc.

Interviewer: What else are they provided?

Respondent: They are also provided meat.

Interviewer: In your opinion, how important is ANC check up to a pregnant woman?

Respondent: I don't know.

Interviewer: Why do you think a pregnant mother and her soon to be born child needs to be examined in ANC check up?

Respondent: I don't know about it.

Interviewer: Whom did you meet while going for the ANC check up?

Respondent: I met the health worker at the health post.

Interviewer: Why did you meet the health worker?

Respondent: Because S/he called me there.

Interviewer: Do you know anyone who doesn't go for the ANC check up?

Respondent: I don't know. I am unsure about it.

Interviewer: Are there any women who do not go to the health post for the ANC check up?

Respondent: No, not really. Most of them go to the health post for the check up.

Interviewer: Where do the women in your village usually go for child delivery?

Respondent: Their first preference for child delivery is health post. If not, they go somewhere else.

Interviewer: Why do they go somewhere else?

Respondent: If they are unable to give birth to their child at the health post, they go somewhere else.

Interviewer: If not health post, where do they exactly go?

Respondent: Some of them go to Kohalpur Medical College, some to Bheri Hospital while some go to Bankatuwa.

Interviewer: Is there anywhere else to go?

Respondent: I don't know. I already told you they go to their first preference is health post.

Interviewer: Why are the women unable to deliver their child at the health post?

Respondent: Maybe because of the weakness of mother or maybe due to some reasons related to the child, I am not sure.

Interviewer: Do you know the signs of danger during pregnancy?

Respondent: No, I don't know.

Interviewer: Where are you planning to deliver your child?

Respondent: I think at the health post.

Interviewer: Why have you thought of delivering at the health post?

Respondent: Because the doctors told me so.

Interviewer: In your opinion, how important is it to have your child delivered at a safe place and by a skilled health worker?

Respondent: I don't know.

Interviewer: Tell me, what could be the importance of it?

Respondent: (Pause)

Interviewer: Tell me the reasons, the need for delivering the child at a safe place.

Respondent: The mother and child will get money and clothes and the child birth process will also be safe and proper.

Interviewer: What are the benefits to the mother?

Respondent: It will also be better for mother.

Interviewer: Do you know the reasons behind women not delivering their child at the health institution?

Respondent: No, I don't know.

Interviewer: What kind of expectations do you have from your husband and your family?

Respondent: Expectations? I didn't get you.

Interviewer: Expectations like; their help and support? Do you have such expectations?

Respondent: I don't know.

Interviewer: In your family, who is responsible for deciding about where you will have your delivery and by whom you will have your child delivered?

Respondent: My husband and mother-in-law decide about it.

Interviewer: What do you think of their decisions? Do you like what they decide for you?

Respondent: Yes.

Interviewer: So, are you happy?

Respondent: Yes, I am.

Interviewer: What kind of preparations have you started doing for your delivery?

Respondent: I have started gathering clothes, storing food and collecting money for my delivery. Since I am the only daughter-in-law of the family, my family love and care for me a lot.

Interviewer: In your opinion, how important is PNC check up after child delivery?

Respondent: It is important.

Interviewer: Why do you think it is important?

Respondent: (Pause) I don't know.

Interviewer: After your delivery, by whom have you thought of getting examined?

Respondent: I have planned to get checked up by the nurse.

Interviewer: Why by the nurse? Do you have any reason?

Respondent: Her attitude and behavior is very good, that's why.

Interviewer: In your community, is the ANC and PNC check up done in a proper way and at the safe and proper place or not?

Respondent: Yes.

Interviewer: Do all the women go for the ANC and PNC check up? Do you know anyone who doesn't?

Respondent: Yes, all of them go for the check up.

Interviewer: Do you know how many times the ANC check up needs to be done?

Respondent: It needs to be done once. Then they will call again on the month of Kartik (November).

Interviewer: Where do you usually go for checkup/treatment when you have any other health related problems?

Respondent: To the Naubasta health post.

Interviewer: Why do you go there?

Respondent: I don't know.

Interviewer: Which is the nearest health center from your house?

Respondent: The one I told you about earlier; Naubasta health post.

Interviewer: How long does it take you to reach this health post from your home?

Respondent: It takes about 30 minutes by walk.

Interviewer: Do you have to face any difficulty while coming to the health post?

Respondent: No.

Interviewer: Do your family member let you visit the health post anytime you want?

Respondent: Yes, first I ask their permission to visit to the health post, they easily let me go.

Interviewer: Do the people of this village have to face any difficulty in getting the access to the health post and the health services provided by it?

Respondent: No, there is no difficulty.

Interviewer: In your family, who is responsible for deciding about where you will go to get the health services?

Respondent: My husband, mother in law, father in law, all of them discuss and decide about it together.

Interviewer: Are you happy with the decisions made by them?

Respondent: Yes, I am. They also consider my choices while making the decision.

Interviewer: So they also ask your opinion?

Respondent: Yes, whatever I ask them for, they try to bring them to me.

Interviewer: What kinds of things do you ask them?

Respondents: I ask them to bring me things like nutritious food, fish & meat, clothes, money etc. and they bring them for me.

Summary:

After the conversation, I concluded that my respondent is illiterate and has inadequate knowledge about ANC, PNC, institutional delivery and their importance. I also found out that she prefers ANC and institutional delivery.
