# Supplementary material for: Maternal health care-seeking behaviour of married adolescent girls: A prospective qualitative study in Banke District, Nepal
Source: PLoS One. 2019 Jun 25;14(6):e0217968. doi: 10.1371/journal.pone.0217968 (PMC6592531; doi:10.1371/journal.pone.0217968)
Supplement: S4 Transcript — (PDF) [file pone.0217968.s007.pdf]

After we finished interviewing Niru Thapa at the VDC office, sister Usha arranged another respondent for us so that we could take her interview. The respondent was waiting inside the hall at the VDC office. I introduced myself and shared about my objectives for coming there with her. After I built a rapport, I started the interview.

Type of the respondent: Pregnant adolescent girl (Married)

Identification of the respondent: -03- Nawabastra

Age of the respondent: 17 years old

Address of the respondent: Bhirawaha - 6, Banke

Duration of pregnancy: 3 months

Date of interview: 1st September 2014, 2071/5/16

Place of interview: VDC's Office ground, Nawabastra, Banke, Nepal

Name of the Interviewer: Sharmila Regmi

Time interview started: -

Time interview completed: -

Interviewer: How old are you?

Respondent: I am 17 years old.

Interviewer: What is your level of education?

Respondent: I have studied till SLC (School leaving certificate). (Means grade 10)

Interviewer: Didn't you study further?

Respondent: No

Interviewer: Why?

Respondent: I gave my SLC examination from Dailekh. Then I got married and came here. I never got back with my studies after that.

Interviewer: You never got back to studying after you got married. Is it because you didn't want to or because your family didn't let you?

Respondent: I myself didn't get back to studying any further.

Interviewer: Why?

Respondent: Just like that. But I have changed my mind now. I have already filled out the form. I will be submitting it soon.

Interviewer: When did you get married?

Respondent: Last year.

Interviewer: You mean at the age of 16?

Respondent: Yes.

Interviewer: How did you get married?

Respondent: I was an arranged marriage. My mother and father arranged it.

Interviewer: What is the occupation of your husband?

Respondent: He has gone abroad to work.

Interviewer: Which country did he go?

Respondent: Dubai.

Interviewer: What is the education level of your husband?

Respondent: He has also studied till SLC.

Interviewer: Do you know what kind of work you husband is doing in Dubai?

Respondent: I don't know.

Interviewer: How is the household expenses managed in your family?

Respondent: It is managed with the help of the money sent by my husband.

Interviewer: How many members are there in your family?

Respondent: My father in law, mother in law, brother in law and his wife and me and my husband. A total of 6 members.

Interviewer: Are you a joint family? Do you all stay together and eat in same kitchen?

Respondent: Yes.

Interviewer: So, is your husband's earning enough to cover you family expenses or are there other income sources?

Respondent: My father in law and brother in law also earn money.

Interviewer: Do they also work? What do they do?

Respondent: My brother in law works at Kohalpur food center and my father in law is a builder.

Interviewer: Did you use any methods of family planning after you got married?

Respondent: No, we didn’t.

Interviewer: When did you get married?

Respondent: Last year in Jestha (May).

Interviewer: And when did your husband go Dubai?

Respondent: Recently. He went there this June.

Interviewer: You got pregnant on the month of Jestha (April-May) and your husband went abroad on Ashad (June) is it?

Respondent: Yes.

Interviewer: Okay, so after you got married, you didn't use any measures of family planning for a year did you?

Respondent: No, we didn't use any.

Interviewer: Do you know anything about the methods of family planning?

Respondent: Yes.

Interviewer: From where did you find out about it?

Respondent: I heard from others and also read about it in books.

Interviewer: So, how did you have the thought of having a child?

Respondent: We never thought about it. Actually, we didn't want to have a baby so soon, but it just happened.

Interviewer: Which one of you didn't want to have a baby soon? You or your husband?

Respondent: Both of us didn't want to have the baby so soon. We had planned to have it a little later.

Interviewer: You could have used measures of family planning if you didn't want it so soon.

Respondent: Well, even if we didn't use it, I didn't get pregnant for a year.

Interviewer: How did you find out that you were pregnant?

Respondent: It was easy to find out about it. I lost my appetite, I felt dizzy and nauseated. I also had the urge to vomit. It was obvious.

Interviewer: But all these symptoms you mentioned are also similar to some other diseases. How were you so sure?

Respondent: I also went to the health post for pregnancy test.

Interviewer: What did you test to confirm your pregnancy?

Respondent: I got my urine tested.

Interviewer: And the health workers there told you about your pregnancy?

Respondent: Yes.

Interviewer: How did you feel when you found out you were going to be a mother soon?

Respondent: How should I be feeling? I felt okay.

Interviewer: Okay as in? Did you feel good, bad, happy or sad when you found it out?

Respondent: I felt happy. I am going to be a mother, I feel happy obviously.

Interviewer: And did you tell your husband after you found out about it?

Respondent: Yes.

Interviewer: When did you tell him?

Respondent: A little later after I found out.

Interviewer: Did you two go to the health post together?

Respondent: No, I went there alone. I told him about it later.

Interviewer: What did your husband say after you told him?

Respondent: What would he say? He said its okay.

Interviewer: Do you know about ANC check up?

Respondent: No, I don’t.

Interviewer: After you became pregnant, have you gone for a checkup?

Respondent: Yes, I went there once. The health worker has told me to come back on the month of Ashwin (September-October).

Interviewer: So, will you go there or not?

Respondent: Yes, I will go.

Interviewer: Do you think it is necessary to go?

Respondent: Yes.

Interviewer: Why do you think so?

Respondent: Because they have called me. So, I have to go.

Interviewer: Do you think you should be going there just because they called you or do you think it's necessary to go for the checkup?

Respondent: I think it is necessary.

Interviewer: Why do you think you should go for the ANC check up?

Respondent: (Silence)

Interviewer: Tell me, why you think you should go for the ANC check up?

Respondent: (Silence)

Interviewer: Why are you silent? Are you scared?

Respondent: No.

Interviewer: Whatever you know about, you can tell me. We have come here to know your views.

Respondent: (Silence)

Interviewer: I think we should go inside, it's very windy here. (The weather outside was terrible, so we went inside the VDC building/office)

Interviewer: Did you have any difficulty in shifting the place?

Respondent: No.

Interviewer: So, tell me now. Why do you think a pregnant woman should go for the ANC check up?

Respondent: (Silence)

Interviewer: Why are you silent? Don’t you know why?

Respondent: No, I don’t know why.

Interviewer: You mentioned earlier that the health worker has called you for the ANC check up on September. When have they called you?

Respondent: They have called me on the 6th of Ashwin i.e. 22nd September.

Interviewer: Why do you think they have called you on 22nd September?

Respondent: (Silent for 10 seconds)

Interviewer: Why do you think the women in your community go for the ANC check up?

Respondent: I don’t know why they go for the check up.

Interviewer: You really don’t know why?

Respondent: Yes.

Interviewer: Do you want to go for the ANC check up or not?

Respondent: Yes, I want to go.

Interviewer: Why do you want to go?

Respondent: (Silent for 15 seconds)

Interviewer: Despite of the scorching heat, why do you want to walk all the way to the health center for check up?

Respondent: (Silent for 5 seconds)

Interviewer: Don’t you know why?

Respondent: I don’t know.

Interviewer: Haven't you heard about it from anyone?

Respondent: No.

Interviewer: Do you think ANC check up of a pregnant woman should be done?

Respondent: Yes, it should be.

Interviewer: Why do you think it should be done?

Respondent: I know that it should be done. But I don’t know why.

Interviewer: It's okay. You can tell whatever you know. By whom do you want to get examined during ANC check up?

Respondent: Where? At the health post?

Interviewer: Yes.

Respondent: I want to get examined by the ANM (Auxiliary Nurse Midwifery) at the health post.

Interviewer: Why do you want to get examined by her?

Respondent: (Silent for 8 seconds)

Interviewer: Why do you want to get examined by the ANM and not somebody else?

Respondent: The ANM has done my check up before as well. That’s why.

Interviewer: Don’t you want to go somewhere else for the checkup?

Respondent: No, we do not go anywhere else except the health post.

Interviewer: Why don't you go somewhere else?

Respondent: The sub health post is the nearest health center for us from our home. So, we go there.

Interviewer: You go to the health post for your ANC check up. Where have you planned to have your child delivered?

Respondent: In the same health post.

Interviewer: Don't you want to go somewhere else for your delivery?

Respondent: (Silent for 5 seconds)

Interviewer: Do you not want to go somewhere else for your delivery or does your family not let you go somewhere else?

Respondent: Why would they let me go? It's my own decision to have my child delivered at the health post.

Interviewer: In your family, who decides where you will go for the ANC check up and where you will have your baby delivered?

Respondent: Nobody in my family decides that.

Interviewer: Why? Doesn't your family discuss with you or suggest you on these matters?

Respondent: Wherever I want to go, I am free to go. That's why.

Interviewer: Okay, so you are free to make your own decisions?

Respondent: Yes.

Interviewer: Whatever you say, your family agrees on that?

Respondent: Yes.

Interviewer: Tell me why have you thought of having your child delivered at the health post, rather than somewhere else?

Respondent: (Silent for 13 seconds)

Interviewer: Come on, tell me. Is it because it's near and easy for you or because the services are good?

Respondent: It's near from here. That's why.

Interviewer: Eventhough you don't know why ANC checkup is important, you know it is needs to be done right? Similarly, do you think it is necessary to go for the checkup after delivery too?

Respondent: After the child birth?

Interviewer: Yes. Is it necessary for you and your child to go for the PNC check up?

Respondent: I don't know. Maybe it is necessary.

Interviewer: Why do you think it is necessary? Do you know? Have you heard anything about it?

Respondent: No.

Interviewer: Please tell me whatever you know about it.

Respondent: (Silent for 12 seconds)

Interviewer: Why aren't you talking? Say something. Have you planned to not speak anything here today?

Respondent: No, it's not like that.

Interviewer: Are you scared?

Respondent: No, why would I be scared?

Interviewer: Do you think it is necessary for the mother and child to go for the PNC check up?

Respondent: (Silent for 8 seconds) I don't know.

Interviewer: You are an educated woman, you must know something. Tell me. Where do you think PNC check up should be done?

Respondent: (After 5 seconds) At the health post?

Interviewer: Do the women in this village go for PNC check up after child delivery?

Respondent: I don't know if they go or not.

Interviewer: Are there any customs/traditions that are being practiced in your family/community after the child birth?

Respondent: (Silence)

Interviewer: Is there the practice of proving dietary supplements, clothes etc. to the mother after delivery?

Respondent: Yes.

Interviewer: What kind of practices are there, tell me.

Respondent: The mothers are given nutritious food to eat while the children get new clothes. These kinds of practices are there.

Interviewer: Whenever you have any health problems, where do you go for checkup/treatment?

Respondent: I go to Bheri hospital.

Interviewer: Okay. So if you have any health problems, you go to Bheri hospital. Whereas, for checkup during pregnancy, you prefer going to your nearest health post is it?

Respondent: No, if the problem can be easily taken care of, I go to the health post. But incase of complications or difficulty, I go to Bheri hospiatal.

Interviewer: What kind of problems do you think can be easily taken care of at the health post and what kind of problems cannot be taken care of there?

Respondent: The health workers will tell me about it. If I they think I will be able to give birth easily at the health post, they will keep me there. Else, they will send me to Bheri hospital.

Interviewer: In what situation do you think they will send you to Bheri hospital?

Respondent: I don't know. The health worker will tell me.

Interviewer: How far is the health post from your home?

Respondent: Not very far. It takes me 10 minutes to get there from my home.

Interviewer: 10 minutes on what?

Respondent: 10 minutes if I go on a bicycle.

Interviewer: How do you usually go to the health post?

Respondent: On the bicycle.

Interviewer: You ride the bicycle during pregnancy too?

Respondent: Yes.

Interviewer: Do you know if riding bicycle during pregnancy can have any negative impact upon unborn child?

Respondent: I don't know.

Interviewer: Do you ride the bicycle alone and go to the health post or do you take somebody else with you?

Respondent: I go there alone.

Interviewer: Do you have to face any difficulty while going to the health post?

Respondent: No.

Interviewer: Do your mother in law and your husband easily let you go to the health post for check up or do you have to face some difficulty?

Respondent: No, I don’t have to face any difficulty.

Interviewer: Do you make decisions for yourself and your child? For example; the decision about where you will be delivering your child?

Respondent: Yes.

Interviewer: How do you feel when you can make your own decisions?

Respondent: I feel good.

Interviewer: Do you earn money?

Respondent: No.

Interviewer: Did you used to work somewhere before?

Respondent: No, I didn’t. I just went to school to study then came back home.

Interviewer: Since you don’t have an income source, when you are in need of something, whom do you ask for money?

Respondent: I ask my mother in law.

Interviewer: And does she give it to you?

Respondent: Yes.

Interviewer: Do you have any difficulty in asking her for money?

Respondent: No.

Interviewer: Do you have any difficulty in going to the health post and getting the services like ANC, PNC check up, child delivery etc.?

Respondent: No.

Interviewer: Where have you thought of delivering your baby?

Respondent: At the Naubastra health post.

Interviewer: Why at this health post?

Respondent: Just like that.

Interviewer: Give me a reason, why at this health post?

Respondent: (Silent for 5 seconds)

Interviewer: Tell me, is it because it's near or because the health workers at the health post are good?

Respondent: Because it's near.

Interviewer: Any other reason?

Respondent: (Pause)

Interviewer: You look as if you know a lot of things, but you don't want to talk much. Is it so?

Respondent: No, it's because I don't know about it.

Interviewer: This is your 1st child right?

Respondent: Yes.

Interviewer: You have decided to deliver your baby at the health post. This is your own decision or has your family suggested it you? Or, had you heard someone say something about delivering the baby at the health post?

Respondent: Yes, I had heard about delivering the baby at the health post.

Interviewer: From whom did you hear about this?

Respondent: From my friends and others.

Interviewer: So, what exactly happens at the health post during delivery?

Respondent: I don’t know.

Interviewer: Where do you think the women in your community usually go for child delivery?

Respondent: Some of the women go to Dusbigha. But, I don’t know where most of the women go, as I have moved to this place recently (a year ago).

Interviewer: Why do you think some women go to Dusbigha instead of going to the nearest health post?

Respondent: (Silent for 5 seconds) Because Dusbigha is better.

Interviewer: Why do you think it is better?

Respondent: Because, even if the child is inappropriately positioned (small/very large sized babies, oppositely positioned body) inside the mother's uterus, the child can be pulled out and delivery can be easy at Dusbigha.

Interviewer: You just mentioned such a nice point. Yes, you must tell me whatever you know okay? I know you know so many things, but you were just scared isn’t it?

Respondent: (Laughs) No, that’s not it.

Interviewer: Where do you want to deliver you child?

Respondent: I will be going to Bheri hospital if there are some complications. If there won't be any, I will deliver at the health post.

Interviewer: Don’t you want to go to Dusbigha?

Respondent: No, I don’t want to.

Interviewer: Why not?

Respondent: Because I went to Bheri hospital instead of Dusbigha for video x-ray services (USG).

Interviewer: You went to the health post to confirm your pregnancy. Where did you go to get your blood tested?

Respondent: To Bheri hospital.

Interviewer: What did you do at the Bheri hospital?

Respondent: I got my blood and urine tested. I also did my USG scan there.

Interviewer: What do you think of the health services at the Bheri hospital?

Respondent: They are okay.

Interviewer: Where do you want to deliver your child now?

Respondent: Like I said earlier, if the child delivery process will be easy and without any complications, I will give birth at the health post, or else, I will go to Bheri.

Interviewer: Do you know what is called easy and which situation/case can be called complicated/ difficult during delifvery?

Respondent: I don’t know. The FCHVs and ANM will let me know about it.

Interviewer: And whatever the ANM suggests you, will you do accordingly?

Respondent: Yes.

Interviewer: That is good.

Interviewer: Do you know anything about the health problems/ issues during pregnancy?

Respondent: What problems/issues?

Interviewer: You might have heard about the complications or health problems that could occur in pregnancy. Do you know about them?

Respondent: No, I don’t know.

Interviewer: Do you think there is the difference between the child delivery experience of an adolescent girl and a mature woman?

Respondent: Yes, I think so.

Interviewer: What do you think are the differences between them?

Respondent: (Silent for 10 seconds)

Interviewer: Do you know anything about it?

Respondent: No.

Interviewer: As an adolescent yourself, what do you want the government and other concerned authorities to do for ensuring proper pregnancy and child delivery related services for the mothers under the age of 19?

Respondent: (Silent for 16 seconds)

Interviewer: Tell me what do you think? Did you understand my question?

Respondent: (Silent for 10 seconds)

Interviewer: What kinds of expectations do you have from your family members during this crucial phase of your life?

Respondent: (Silent for 7 seconds)

Interviewer: Do you not want to talk?

Respondent: (Silent for 5 seconds)

Interviewer: Are you in a hurry to go somewhere from here?

Respondent: No, there is no hurry.

Interviewer: Are you having any difficulty in talking to me?

Respondent: (Silent for 10 seconds)

Interviewer: Are you bored?

Respondent: No.

Interviewer: How difficult and uneasy do you feel after getting pregnant?

Respondent: It is a little uneasy.

Interviewer: At times, don’t you feel like vomiting?

Respondent: Yes, I do.

Interviewer: During times like this, how do you wish your family supported you?

Respondent: (Silent for 8 seconds)

Interviewer: Don’t you want them to love and care for you and support you in your work?

Respondent: No.

Interviewer: Why not?

Respondent: (Silent for 10 seconds)

Interviewer: Why have you stopped responding to my questions sister?

Respondent: (Silent for 10 seconds)

Interviewer: Do you know what kinds of planning and preparations must be done by the pregnant woman and her family before the child delivery?

Respondent: No, I don’t know.

Interviewer: Don’t you think preparation of food, clothes and arrangement for money incase of emergency is necessary to be done before child delivery?

Respondent: (Silent for 3 seconds)

Interviewer: Do you understand what I am saying?

Respondent: Yes. I don’t think such preparation is necessary because there are people in my family to take care of those things when it's necessary. If there

wasn’t anybody else in my family, maybe I would have to be doing such preparations.

Interviewer: You having been talking much in this conversation, is it really because you didn’t know about the things I asked you?

Respondent: Yes, it's because I don’t know.

Interviewer: The health worker has called you for the ANC checkup on the 22nd Ashwin (October 6) right? Will you be going there?

Respondent: Yes.

Interviewer: Why do you think you must be going for the ANC check up?

Respondent: (Silence)

Interviewer: You have studied till grade 10. Don’t you know why you should go for the check up?

Respondent: (Silence)

Interviewer: Do the women in this village go for the ANC/PNC checkup?

Respondent: Maybe. I am not sure.

Interviewer: Are you new in this village?

Respondent: Yes. I am from Dailekh. A year ago, I got married and then came here.

Interviewer: That's why don’t know much about this village. How far is the health post from your home at Dailekh?

Respondent: It is very near.

Interviewer: Did you go to the health post there?

Respondent: No, there wasn’t any reason for me to visit the health post.

Interviewer: Since your pregnancy, you have gone for the ANC check up only once right?

Respondent: Yes.

Interviewer: Where did you go for the check up at that time?

Respondent: To the health post.

Interviewer: And why did you go to Bheri hospital?

Respondent: The ANM from the health post told me to do the USG scan at Bheri hospital. She also told me to get my blood tested there and then bring her its report. So, I went there.

Interviewer: Where do you think women in this village go for the ANC check up?

Respondent: To the health post.

Interviewer: Where have you thought of going to for the ANC check up?

Respondent: To the health post.

Interviewer: Why to the health post?

Respondent: Because it's near from my home.

Interviewer: If you have any query, you can ask them. Thank you so much for you time.

Memo:

I wonder how my respondent studied till SLC. She doesn’t know about the ANC and PNC care. May be she didn’t know about it because she is an adolescent and she used to live in Dailekh (Dailekh lies in hilly region of Nepal).
